# Cross-national prevalence of mental disorders in older adults exposed to COVID-19 information*

**DOI:** 10.1590/1518-8345.7580.4632

**Published:** 2025-08-18

**Authors:** Daniele Knopp Ribeiro, Fábio da Costa Carbogim, Patricia Rodrigues Braz, Sofia Sabina Lavado-Huarcaya, Aracely Díaz-Oviedo, Alexandre Favero Bulgarelli, Rosimere Ferreira Santana, Ione Carvalho Pinto, Fabiana Costa Machado Zacharias, Ricardo Bezerra Cavalcante

**Affiliations:** 1 Universidade Federal de Juiz de Fora, Juiz de Fora, MG, Brazil.; 2 Scholarship holder at the Coordenação de Aperfeiçoamento de Pessoal de Nível Superior (CAPES), Brazil.; 3 Universidad Señor de Sipán, Escuela de Postgrado, Pimentel, Lambayeque, Peru.; 4 Universidad Autónoma de San Luís Potosí, Facultad de Enfermería y Nutrición, San Luis Potosí, SLP, Mexico.; 5 Universidade Federal do Rio Grande do Sul, Porto Alegre, RS, Brazil.; 6 Universidade Federal Fluminense, Faculdade Enfermagem, Niterói, RJ, Brazil.; 7 Universidade de São Paulo, Escola de Enfermagem de Ribeirão Preto, PAHO/WHO Collaborating Centre for Nursing Research Development, Ribeirão Preto, SP, Brazil.

**Keywords:** Infodemic, Mental Health, Aged, Latin America, Covid-19, Cross-Sectional Studies

## Abstract

to verify the association between exposure to COVID-19 news and information through social networks, television and radio, as well as to screen for geriatric anxiety and depression comparing Peru, Brazil and Mexico.

a cross-sectional design, web-based survey with non-probability sampling and validated scales to screen for geriatric anxiety and depression, as well as data analysis by hierarchical binary logistic regression.

there was prevalence of female gender (n=4,937; 61.9%), non-white race/skin color (n=4,724; 59.2%) and age group of 60 to 64 years old (n=2,584; 32.4%) among the 7,976 participants. COVID-19 news and information were accessed through television (n=6,187;77.6%), a few or some times a week (n=4,322, 54.2%) and for at least three hours (n=2,596; 32.5%). In the final models and both for anxiety and for depression, the significant differences (p-value<0.001) for the “use”, “exposure frequency” and “exposure hours” aspects changed depending on the media. The prevalence of the outcomes in the three countries was low.

frequent exposure to media was associated with higher prevalence of geriatric anxiety and depression, although the difference across the countries under study was small.

## Highlights

**(1)** The variables associated with the outcomes indicate consumption of news and information. **(2)** Media exposure frequency and time matter for screening. **(3)** The prevalence of the outcomes when comparing all three countries was small. **(4)** Screening was 42.6% for anxiety and 59.4% for depression. **(5)** Media consumption was 77.6% television, 46.6% social media and 42.7% radio.

## Introduction

On January 30^th^, 2020, and later on March 11^th^ of the same year, the risk to public health and the dissemination capacity of the SARS-CoV-2 coronavirus (responsible for COVID-19) were recognized. Approximately four years later, more than 772 million cases and approximately 7 million deaths have been reported worldwide, with the European and Americas regions standing out in prevalence of reported cases and deaths, respectively^(1)^.

The Latin American and Caribbean population health has been the most impacted by the COVID-19 pandemic in the world. Despite the different arrangements of health systems and social/political diversity, historically vulnerable groups such as women, non-white skinned individuals, people living in poverty and/or on the streets, students and the elderly experienced the pandemic differently and were affected by the intensification of historical inequalities in the region^(2-3)^.

The complexity of the pandemic experience was increased by excess and speed of the information produced and disseminated in traditional mass media and was further amplified by social media. In this context, recognizing the information origin, intention and quality demands time, a phenomenon that is called infodemic^(4)^. This can result in changes in risk perception, feelings and sensations of confusion and disorientation, hesitation, paralysis, denial and distrust, among others, which are related to people’s previous experiences and with trusted individuals, the health system and the government of the region^(5-6)^.

Less than a year after the pandemic was declared, the incipient structure and coordination of Latin American governments in managing the infodemic was reported^(7)^, based on an analysis of the websites of the ministries of health from 10 countries. In parallel^(8)^, in a descriptive ecological study it was highlighted that, in six Latin American countries researched, the places with the greatest difficulty recognizing fake news and the centrality of social networks as an information means coincided with the highest mortality rates due to COVID-19 during that period.

Furthermore, in the Brazilian and Chilean news media in 2020/2021, it was possible to find “they’ve already lived their lives” discourses, blaming the elderly for the severity of the pandemic and the homogeneity of deaths, when referring to aged people. In turn, the conceivable mental health deterioration after the pandemic is also recognized, resulting from the ridicule, abandonment, dependence and control to which this population segment was subjected^(9)^.

Older people’s higher vulnerability is a result of the historical socioeconomic and demographic inequalities associated with the known increase in population aging, which is evident in the mortality rate proportional to age increase during the COVID-19 pandemic^(1-2)^. The need for research, care and strengthening of public policies is part of the movement for the 2021-2030 Decade of Healthy Aging, coordinated by the Pan American Health Organization (PAHO)^(10)^ in the Americas.

The current research is justified given the absence of studies with populations exclusively comprised by aged people and/or the implications for mental health resulting from the infodemic in Latin American countries such as Peru, Brazil and Mexico. Even when considering that complex symptoms such as anxiety, depression and insomnia during the pandemic are reported in systematic reviews with the adult population in different world regions, the focus is mainly on social networks when associated with the media^(11-13)^.

Thus, the objective is to verify the association between exposure to COVID-19 news and information through social networks, television and radio, as well as to screen for geriatric anxiety and depression, comparing aged people from Peru, Brazil and Mexico.

## Method

### Study design

A cross-sectional study conducted between July 2020 and June 2022 with aged people over 60 years old in Peru, Brazil and Mexico. It derives from Part 1 of the research entitled “The COVID-19 Infodemic and its repercussions on aged people’s mental health during and after the pandemic: A multicenter study in Brazil, Peru, Colombia, Mexico and Portugal”, aimed at “analyzing the relationship between the COVID-19 infodemic and its repercussions on aged people’s mental health”.

### Ethical aspects

The study was approved by the Human Research Ethics Committees in Brazil (CAAE: 31932620.1.1001.5147, Opinion No. 4,134,050), Peru and Mexico (Autonomous University of San Luis Potosí, registration code: CONBIOÉTICA-24-CEI-002-20230925).

### Selection criteria

The subjects included were aged individuals over 60 years old with preserved cognitive ability, access to email and/or social networks and/or telephone. Older adults who were unable to answer the questions on their own according to the self-report of the person contacted were excluded.

### Loci

The participants lived in the following cities: Brazil - Brasilia, Divinópolis, Juiz de Fora, Porto Alegre, Rio de Janeiro, Ribeirão Preto, São Paulo and Viçosa; Mexico - Cuidad Valles, Matehuala, Rioverde, Salinas de Hidalgo, San Luis Potosí, Soledas de Graciano Sánchez and Tamazunchale; Peru - Arequipa, Cerro de Pasco, Chiclayo, Huánuco, Iquitos, Lima, Puno-Juliaca, Tacna, Tarapoto, Tumbes and Trujillo.

### Data collection

Data collection took place via a web-based survey with non-probability sampling, distributed via email and social networks (WhatsApp, Facebook and Instagram) and announced by Geriatrics and Gerontology scientific societies, health care institutions, retirement associations and research centers. A total of 15 pilot interviews were conducted in each country to adapt the language to the questions.

The link to access the questionnaire directed to a Free and Informed Consent Form (FICF). Only participants who agreed to the question had access to it, and all of them were marked as mandatory. Everyone was sent an FICF copy signed by the researcher via email or social media. The elderly were also contacted by telephone with an invitation to participate in the research at the time of the call or when rescheduling, with them signing the FICF and indicating their agreement to recording the call. The FICF signed by the researcher was later also sent via email or social media.

### Instruments

The questionnaire consisted of the following segments: Identification, Sociodemographic profile, Exposure to COVID-19 news and information^(14-15)^, Impact of signs and symptoms on psychopathological changes when exposed to COVID-19 information (research in development), Perceived Stress Scale, Geriatric Anxiety Inventory (GAI)^(16)^ and Geriatric Depression Scale (GDS-15)^(17)^. The current study analyzes the Sociodemographic profile, Exposure to COVID-19 news and information, GAI and GDS-15 segments^(16-17)^.

For the data collected in Brazil, the GAI^(18)^ scale validated with aged people in the country consists of 20 self-report items with dichotomous questions (I agree/I disagree) and cutoff points of <13 for non-cases and ≥13 for cases. Also validated, GDS-15^(19)^, contains 15 questions, of which 10 are scored if answered positively and the others are answered negatively, with cutoff points of <6 for non-cases and ≥6 for cases.

The data collected in Mexico follow GAI^(20)^, a version validated in Spanish with aged people from Madrid given the absence of a validation study in the country. The inventory consists of 20 dichotomous items (I agree/I disagree) with cutoff points of ≥11 for cases and <11 for non-cases. Validated with aged Mexican people^(21)^, GDS-15 consists of 15 questions with Yes/No options in which 10 are scored if answered positively and the others if answered negatively. The authors do not indicate any cutoff point; a comparison with high scores (10 or more symptoms) and low scores (less than five symptoms) was made for depression. Thus, cases correspond to ≥5 and non-cases to <5.

GAI^(20)^ was also followed in the case of the data from Peru. The version used was the one validated in Spanish with aged people from Madrid given the absence of a validation study in the country. The inventory consists of 20 dichotomous items (I agree/I disagree) with cutoff points of ≥11 for cases and <11 for non-cases. When assessing depression symptoms, the reference validated for aged people in Colombia was used, consisting of 15 dichotomous Yes/No items, in which 10 questions are scored if answered positively and the others if answered negatively; the proposed cutoff point was ≥5 for suggested depression^(22)^.

The independent variables related to the outcomes were organized into the following hierarchical blocks of the analysis model:

Block 1 - Participants’ origin variable: Peru, Brazil and Mexico.

Block 2 - Socioeconomic and demographic variables: gender; age group; marital status; race/skin color; lives with; housing situation; area of residence; maximum schooling level; change in income due to the COVID-19 pandemic.

Block 3 - Exposure to COVID-19 news and information variables: type of media; exposure frequency in the last week; exposure hours.

### Data treatment and analysis

In the analysis of the data collected, the participants were assigned a number code to preserve confidentiality; the tabulation was developed in Google Sheets and imported into the IBM Statistical Package for the Social Sciences (SPSS) software, version 23.0 for Windows. All variables were subjected to descriptive analysis, calculating absolute (n) and relative (%) frequency distributions. To analyze the associations, Pearson’s chi-square (X^2^) test of independence was used in the bivariate analysis corresponding to each block for the nominal categorical independent variables; in turn, Mann-Whitney’s U test was employed for the ordinal independent variables.

This was followed by a hierarchical binary logistic regression analysis in which all variables in the respective blocks initially produced analyses separately. The variables were introduced into the final multivariate model for comparison and according to block of origin. A 5% significance level and 95% confidence intervals were considered for all tests.

## Results

Of all 7,976 participants, 4,377 (54.9%) were from Peru, 3,307 (41.5%) from Brazil and 292 (3.7%) from Mexico. In this sample, 4,937 (61.9%) stated being female and 4,724 (59.2%) reported belonging to non-white races/skin colors, including black, brown, Asian and indigenous ancestry. The most frequent age group was between 60 and 64 years old, with 2,584 (32.4%) older adults. Regarding marital status, 4,642 (58.2%) aged individuals stated being married or living with a partner.

As for housing, 6,514 (81.7%) reported owning their own house, 6,830 (85.6%) lived in urban areas and 3,744 (46.9%) lived with at least three people in the same house. Regarding the highest schooling level indicated, Higher Education was the most frequent with 2,120 (26.6%) aged subjects. In relation to the influence of the COVID-19 pandemic on income, 4,112 (51.6%) participants considered that it remained unchanged.

Regarding COVID-19 information on social media, 4,259 (53.4%) participants stated not using them and 3,261 (40.9%) reported no exposure in the week prior to the survey.

In the case of 6,187 (77.6%) participants, television (TV) was used to access COVID-19 news and information; however, 4,322 (54.2%) reported watching TV a few or some times in the week prior to taking part in the survey. For 2,596 (32.5%) older adults, TV use was in the range of three or more hours per week.

Regarding radio use, 4,568 (57.3%) subjects reported not having accessed COVID-19 news and information at all through this medium in the week prior to participating in the survey. The data tabulated by country can be seen in Table 1 below:

**Table 1-:** Frequency values according to socioeconomic and demographic characteristics and exposure to COVID-19 news and information among older adults (n* = 7,976). Peru, Brazil, Mexico, 2022

**Variables**	**Countries**							
	**Peru** **Frequency** **(n=4,377)** ^†^		**Brazil** **Frequency** **(n=3,307)** ^‡^		**Mexico** **Frequency** **(n=292)** ^§^		**Total** **Frequency** (n=7,976)*	
	**N** ^||^	**%** ^¶^	**N** ^||^	**%** ^¶^	**N** ^||^	**%** ^¶^	**N** ^||^	**%** ^¶^
**Block 2 - Socioeconomic and demographic variables**								
**Biological sex**								
Female	2,452	56.0	2,250	68.0	239	81.8	4,941	61.9
Male	1,925	44.0	1,039	31.4	53	18.2	3,017	37.8
I prefer not to answer	0	0.0	18	0.5	0	0.0	18	0.2
**Age group (years)**								
60-64	1,241	28.4	1,285	38.9	61	20.9	2,587	32.4
65-69	1,284	29.3	921	27.9	86	29.5	2,291	28.7
70-74	705	16.1	503	15.2	60	20.5	1,268	15.9
75-79	547	12.5	334	10.1	43	14.7	924	11.6
80+	600	13.7	264	8.0	42	14.4	906	11.4
**Marital status**								
Single	423	9.7	365	11.0	36	12.3	824	10.3
Married/Living together	2,670	61.0	1,835	55.5	137	46.9	4,642	58.2
Separated/Divorced	322	7.4	509	15.4	23	7.9	854	10.7
Widowed	962	22.0	598	18.1	96	32.9	1,656	20.8
**Race/Skin color**								
White	607	13.9	2,364	71.5	281	96.2	3,252	40.8
Non-white	3,770	86.1	943	28.5	11	3.8	4,724	59.2
**Number of people living in the same house**								
Lives alone	189	4.3	587	17.8	33	11.3	809	10.1
One or two	1,412	32.3	1,886	57.0	125	42.8	3,423	42.9
Three or more	2,776	63.4	834	25.2	134	45.9	3,744	46.9
**Own house**								
No	872	19.9	551	16.7	39	13.4	1,462	18.3
Yes	3,505	80.1	2,756	83.3	253	86.6	6,514	81.7
**Area of residence**								
Urban	3,380	77.2	3,160	95.6	290	99.3	6,830	85.6
Rural	997	22.8	147	4.4	2	0.7	1,146	14.4
**Maximum schooling level**								
Did not study or Incomplete Basic Education	355	8.1	295	8.9	25	8.6	675	8.5
Basic Education or Elementary School	1,133	25.9	713	21.6	116	39.7	1,962	24.6
High School	1,305	29.8	718	21.7	56	19.2	2,079	26.1
Complete Higher Education	1,446	33.0	645	19.5	29	9.9	2,120	26.6
Others	138	3.2	936	28.3	66	22.6	1,140	14.3
**Change in income during the COVID-19 pandemic**								
No	1,536	35.1	2,437	73.8	139	47.6	4,112	51.6
Yes, it increased	172	3.9	80	2.4	15	5.1	267	3.3
Yes, it decreased	2,669	61.0	787	23.8	138	47.3	3,594	45.1
**Block 3 - Exposure to COVID-19 news and information variables**								
Uses social media to access COVID-19 news and information* *								
No	2,719	62.1	1,361	41.2	179	61.3	4,259	53.4
Yes	1,658	37.9	1,943	58.8	113	38.7	3,714	46.6
**Exposure frequency to COVID-19 news and information on social media in the last week**								
Not once	2,280	52.1	822	24.9	159	54.5	3,261	40.9
A few or some times	1,571	35.9	1,464	44.3	102	34.9	3,137	39.3
Frequently	526	12.0	1,021	30.9	31	10.6	1,578	19.8
Exposure hours to COVID-19 news and information on social media**								
0	2,097	47.9	848	25.7	182	62.3	3,127	39.2
1	1,080	24.7	811	24.6	49	16.8	1,940	24.3
2-5	1,004	22.9	1,084	32.8	58	19.9	2,146	26.9
6+	196	4.5	560	17.0	3	1.0	759	9.5
Uses television to access COVID-19 news and information**								
No	1,002	22.9	624	18.9	160	54.8	1,786	22.4
Yes	3,375	77.1	2,680	81.1	132	45.2	6,187	77.6
**Exposure frequency to COVID-19 news and information on television in the last week**								
Not once	601	13.7	394	11.9	41	14.0	1,036	13.0
A few or some times	2,669	61.0	1,440	43.5	213	72.9	4,322	54.2
Frequently	1,107	25.3	1,473	44.5	38	13.0	2,618	32.8
Exposure hours to COVID-19 news and information on television**								
0	597	13.6	431	13.1	53	18.2	1,081	13.6
1	1,595	36.4	884	26.8	111	38.0	2,590	32.5
2	947	21.6	686	20.8	71	24.3	1,704	21.4
3+	1,238	28.3	1,301	39.4	57	19.5	2,596	32.5
Uses radio to access COVID-19 news and information**								
No	1,866	42.6	2,429	73.5	273	93.5	4,568	57.3
Yes	2,511	57.4	876	26.5	19	6.5	3,406	42.7
**Exposure frequency to COVID-19 news and information on the radio in the last week**								
Not once	1,317	30.1	1,956	59.1	211	72.3	3,484	43.7
A few or some times	2,333	53.3	956	28.9	65	22.3	3,354	42.1
Frequently	727	16.6	395	11.9	16	5.5	1,138	14.3
Exposure hours to COVID-19 news and information on the radio**								
0	1,330	30.4	2,083	63.0	223	76.4	3,636	45.6
1+	3,047	69.6	1,223	37.0	69	23.6	4,339	54.4

*(n=7,976) = Study population; ^†^Total sample from Peru; ^‡^Total sample from Brazil; ^§^Total sample from Mexico; ^||^N = Distribution of participants in each answer; ^¶^% = Percentage in relation to the total answers in the column corresponding to each variable; **Total number of respondents lower than total study population (7,976)

Of the 7,976 participating aged individuals, 3,395 (42.6%) reached the screening cutoff point for geriatric anxiety, while 4,581 (57.4%) did not. In the bivariate analysis corresponding to the socioeconomic and demographic variables (Block 2), the following items obtained significant differences, with p-value<0.001: biological sex, age group, country of origin, marital status, race/skin color, number of people living in the same house, area of residence and change in income during the COVID-19 pandemic; as was also the case in the “maximum schooling level” item, with p-value=0.032 (Table 2 and Table 3).

**Table 2-:** Screening/Not screening for geriatric anxiety and depression and p-values* corresponding to socioeconomic and demographic characteristics and exposure to COVID-19 news and information among older adults (n^†^ = 7,976). Peru, Brazil, Mexico, 2022

**Variables**	**Screening for geriatric anxiety**				p*	**Screening for geriatric depression**				p*
	**No**		**Yes**			**No**		**Yes**		
	**N** ^‡^	**%** ^§^	**N** ^‡^	**%** ^§^		**N** ^‡^	**%** ^§^	**N** ^‡^	**%** ^§^	
**Block 1 - Participants’ origin variable and Block 2 - Socioeconomic and demographic variables**										
**Biological sex**										
Female	2,985	65.2	1,956	57.6	<0.001	2,036	62.8	2,905	61.4	0.434
Male	1,584	34.6	1,433	42.2		1,199	37.0	1,818	38.4	
I prefer not to answer	12	0.3	6	0.2		7	0.2	11	0.2	
**Country of origin**										
Peru	1,599	34.9	2,778	81.8	<0.001	1,134	35.0	3,243	68.5	<0.001
Brazil	2,712	59.2	595	17.5		1,987	61.3	1,320	27.9	
Mexico	270	5.9	22	0.6		121	3.7	171	3.6	
**Marital status**										
Single	434	9.5	390	11.5	<0.001	352	10.9	472	10.0	<0.001
Married/Living together	2,662	58.1	1,980	58.3		1,842	56.8	2,800	59.1	
Separated/Divorced	552	12.0	302	8.9		432	13.3	422	8.9	
Widowed	933	20.4	723	21.3		616	19.0	1,040	22.0	
**Race/Skin color**										
White	2,492	54.4	760	22.4	<0.001	1,674	51.6	1,578	33.3	<0.001
Non-white	2,089	45.6	2,635	77.6		1,568	48.4	3,156	66.7	
**Own house**										
No	828	18.1	634	18.7	0.501	549	16.9	913	19.3	0.008
Yes	3,753	81.9	2,761	81.3		2,693	83.1	3,821	80.7	
**Area of residence**										
Urban	4,050	88.4	2,780	81.9	<0.001	2,920	90.1	3,910	82.6	<0.001
Rural	531	11.6	615	18.1		322	9.9	824	17.4	
**Block 3 - Exposure to COVID-19 news and information variables**										
**Uses social media to access COVID-19 news and information**										
No	2,300	50.2	1,959	57.7	<0.001	1,613	49.8	2,646	55.9	<0.001
Yes	2,278	49.8	1,436	42.3		1,627	50.2	2,087	44.1	
**Uses television to access COVID-19 news and information**										
No	1,060	23.2	726	21.4	0.061	690	21.3	1,096	23.2	0.052
Yes	3,518	76.8	2,669	78.6		2,550	78.7	3,637	76.8	
**Uses radio to access COVID-19 news and information**										
No	2,877	62.8	1,691	49.8	<0.001	2,111	65.2	2,457	51.9	<0.001
Yes	1,702	37.2	1,704	50.2		1,129	34.8	2,277	48.1	

*p = Pearson’s chi-square test; ^†^Study population; ^‡^N = Distribution of participants in each answer; ^§^% = Percentage in relation to the total answers in the column referring to each variable

**Table 3-:** Screening/Not screening for geriatric anxiety and depression and p-values* corresponding to socioeconomic and demographic characteristics and exposure to COVID-19 news and information among older adults (n^†^ = 7,976). Peru, Brazil, Mexico, 2022

**Variables**	**Screening for geriatric anxiety**				p*	**Screening for geriatric depression**				p*
	**No**		**Yes**			**No**		**Yes**		
	**N** ^‡^	**%** ^§^	**N** ^‡^	**%** ^§^		**N** ^‡^	**%** ^§^	**N** ^‡^	**%** ^§^	
**Block 1 - Participants’ origin variable and Block 2 - Socioeconomic and demographic variables**										
**Age group (years)**										
60-64	1,570	34.3	1,017	30.0	<0.001	1,191	36.7	1,396	29.5	<0.001
65-69	1,303	28.4	988	29.1		889	27.4	1,402	29.6	
70-74	730	15.9	538	15.8		514	15.9	754	15.9	
75-79	507	11.1	417	12.3		358	11.0	566	12.0	
80+	471	10.3	435	12.8		290	8.9	616	13.0	
**Number of people living in the same house**										
Lives alone	569	12.4	240	7.1	<0.001	427	13.2	382	8.1	<0.001
One or two	2,155	47.0	1,268	37.3		1,520	46.9	1,903	40.2	
Three or more	1,857	40.5	1,887	55.6		1,295	39.9	2,449	51.7	
**Maximum schooling level**										
Did not study or Incomplete Basic Education	403	8.8	272	8.0	0.032	234	7.2	441	9.3	<0.001
Basic Education or Elementary School	1,163	25.4	799	23.5		751	23.2	1,211	25.6	
High School	1,135	24.8	944	27.8		807	24.9	1,272	26.9	
Complete Higher Education	1,029	22.5	1.091	32.1		784	24.2	1,336	28.2	
Others	851	18.6	289	8.5		666	20.5	474	10.0	
**Change in income during the COVID-19 pandemic**										
No	2,564	56.0	1,548	45.6	<0.001	1,977	61.0	2,135	45.1	<0.001
Yes, it increased	149	3.3	118	3.5		105	3.2	162	3.4	
Yes, it decreased	1,866	40.8	1,728	50.9		1,158	35.7	2,436	51.5	
**Block 3 - Exposure to COVID-19 news and information variables**										
**Exposure frequency to COVID-19 news and information on social media in the last week**										
Not once	1,686	36.8	1,575	46.4	<0.001	1,146	35.3	2,115	44.7	<0.001
A few or some times	1,913	41.8	1,224	36.1		1,325	40.9	1,812	38.3	
Frequently	982	21.4	596	17.6		771	23.8	807	17.0	
**Exposure hours to COVID-19 news and information on social media**										
0	1,635	35.7	1,492	43.9	<0.001	1,118	34.5	2,009	42.4	<0.001
1	1,059	23.1	881	25.9		836	25.8	1,104	23.3	
2-5	1,368	29.9	778	22.9		930	28.7	1,216	25.7	
6+	515	11.3	244	7.2		355	11.0	404	8.0	
**Exposure frequency to COVID-19 news and information on television in the last week**										
Not once	572	12.5	464	13.7	<0.001	421	13.0	615	13.0	<0.001
A few or some times	2,414	52.7	1,908	56.2		1,662	51.3	2,660	56.2	
Frequently	1,595	34.8	1,023	30.1		1,159	35.7	1,459	30.8	
**Exposure hours to COVID-19 news and information on television**										
0	590	12.9	491	14.5	0.012	479	14.8	602	12.7	0.075
1	1,501	32.8	1,089	32.1		1,028	31.7	1,562	33.0	
2	924	20.2	780	23.0		708	21.9	996	21.0	
3+	1,561	34.1	1,035	30.5		1,024	31.6	1,572	33.2	
**Exposure frequency to COVID-19 news and information on the radio in the last week**										
Not once	2,180	47.6	1,304	38.4	<0.001	1,656	51.1	1,828	38.6	<0.001
A few or some times	1,838	40.1	1,516	44.7		1,190	36.7	2.164	45.7	
Frequently	563	12.3	575	16.9		396	12.2	742	15.7	
**Exposure hours to COVID-19 news and information on the radio**										
0	2,295	50.1	1,341	39.5	<0.001	1,779	54.9	1,857	39.2	<0.001
1+	2,285	49.9	2,054	60.5		1,462	45.1	2,877	60.8	

*p = Mann-Whitney’s U test; ^†^Study population; ^‡^N = Distribution of participants in each answer; ^§^% = Percentage in relation to the total answers in the column referring to each variable

In the association of the screening/not screening for anxiety variable with the exposure to COVID-19 news and information variables (Block 3), significant differences were observed in the following items: use, exposure frequency and exposure hours through social networks to access COVID-19 news and information, with p-values<0.001. In the case of use, exposure frequency and exposure hours through radio to access COVID-19 news and information, p-value<0.001 was obtained. Regarding TV, only the “exposure frequency” and “exposure hours” variables were significant, with p-value<0.001 and p-value=0.012, respectively.

In terms of screening for geriatric depression, 4,734 (59.4%) indicated it, while 3,242 (40.6%) did not. The following socioeconomic and demographic variables (Blocks 1 and 3) were significant in the bivariate analysis: age group, country of origin, marital status, race/skin color, number of people living in the same house, area of residence, maximum schooling level and change in income during the COVID-19 pandemic, with p-value<0.001 for each item. Among the significant differences, only the “own house” item reached p-value=0.008.

From the bivariate association analysis between screening/not screening for geriatric depression and the exposure to COVID-19 news and information variables (Block 3), the following items were statistically significant: use, exposure frequency and exposure hours through social networks to access COVID-19 news and information, with p-value<0.001 for each item. In the case of use, exposure frequency and exposure hours through radio to access COVID-19 news and information, p-values<0.001 were respectively obtained. In relation to TV, only the “frequency” item was significant, with p-value<0.001.

In the multivariate analysis, the following variables presented p-values<0.05 in the independent regression corresponding to the blocks for anxiety screening: Block 1, country of origin; Block 2, gender, age group, marital status, race/skin color (non-white), number of people living in the same house, area of residence, maximum schooling level (from High School upwards) and change in income during the COVID-19 pandemic (decrease in income); Block 3, exposure frequency (some times) and exposure hours (at least two) to COVID-19 news and information on social networks, use and exposure hours (one) to COVID-19 news and information on television, and use, exposure frequency (frequently) and no exposure hours to COVID-19 news and information on the radio.

However, in the final model (Table 4) and with Nagelkerke’s R²=0.312 and p-value<0.001 as per Hosmer-Lemeshow test, the following variables remained significant: country of origin, gender (male), marital status, race/skin color (non-white), change in income during the COVID-19 pandemic, exposure frequency (frequently) and exposure hours (at least two) to COVID-19 news and information on social media, television use and hours (one) to access COVID-19 news and information, and exposure frequency (some times) to COVID-19 news and information on the radio.

**Table 4-:** Final model of screening/not screening for geriatric anxiety and p-values* corresponding to socioeconomic and demographic characteristics and exposure to COVID-19 news and information among older adults (n^†^ = 7,976). Peru, Brazil, Mexico, 2022

**Variables**	**Exposition (B) adjusted per block (95% CI) ^‡^**	p*	**Exposition (B) adjusted in the final model (95% CI)** ^‡^	p*
**Block 1 - Participants’ origin variable**				
**Country of origin**				
Peru	Ref ^§^			
Brazil	0.13	<0.001	0.10	<0.001
	0.11-0.14		0.09-0.12	
Mexico	0.05	<0.001	0.05	<0.001
	0.03-0.07		0.03-0.08	
**Block 2 - Socioeconomic and demographic variables**				
**Biological sex**				
Female	Ref ^§^			
Male	1.13	0.018	1.13	0.031
	1.02-1.25		1.01-1.26	
I prefer not to answer	0.85	0.761	2.46	0.079
	0.31-2.38		0.90-6.72	
**Age group (years)**				
60-64	Ref ^§^			
65-69	1.14	0.043	1.07	0.300
	1.00-1.29		0.94-1.23	
70-74	1.18	0.031	0.99	0.900
	1.02-1.37		0.84-1.16	
75-79	1.26	0.006	1.05	0.577
	1.07-1.49		0.88-1.27	
80+	1.45	<0.001	1.04	0.690
	1.22-1.73		0.86-1.26	
**Marital status**				
Single	Ref ^§^			
Married/Living together	0.61	<0.001	0.68	<0.001
	0.52-0.73		0.57-0.82	
Separated/Divorced	0.63	<0.001	0.74	0.009
	0.51-0.78		0.59-0.93	
Widowed	0.70	<0.001	0.72	0.002
	0.58-0.84		0.59-0.89	
**Race/Skin color**				
White	Ref ^§^			
Non-white	3.63	<0.001	1.39	<0.001
	3.26-4.05		1.21-1.59	
**Number of people living in the same house**				
Lives alone	Ref ^§^			
One or two	1.26	0.017	1.06	0.563
	1.04-1.51		0.87-1.30	
Three or more	1.76	<0.001	1.02	0.886
	1.45-2.12		0.82-1.25	
**Own house**				
No	Ref ^§^			
Yes	1.10	0.146	1.13	0.073
	0.97-1.24		0.99-1.30	
**Area of residence**				
Urban	Ref ^§^			
Rural	1.34	<0.001	0.92	0.257
	1.17-1.54		2.79-1.06	
**Maximum schooling level**				
Did not study or Incomplete Basic Education	Ref ^§^			
Basic Education or Elementary School	1.15	0.139	0.92	0.420
	0.95-1.39		0.75-1.13	
High School	1.60	<0.001	1.04	0.754
	1.32-1.94		0.83-1.28	
Complete Higher Education	2.21	<0.001	1.22	0.082
	1.82-2.68		0.97-1.53	
Others	1.28	0.039	1.21	0.144
	1.01-1.61		0.94-1.58	
**Change in income during the COVID-19 pandemic**				
No	Ref ^§^			
Yes, it increased	1.06	0.671	0.71	0.021
	0.81-1.39		0.53-0.95	
Yes, it decreased	1.12	0.029	0.72	<0.001
	1.01-1.24		0.64-0.81	
**Block 3 - Exposure to COVID-19 news and information variables**				
**Uses social media to access COVID-19 news and information about**				
No	Ref ^§^			
Yes	1.05	0.448	0.98	0.819
	0.92-1.20		0.85-1.14	
**Exposure frequency to COVID-19 news and information on social media in the last week**				
Not once	Ref ^§^			
A few or some times	0.77	0.002	1.13	0.217
	0.65-0.91		0.93-1.36	
Frequently	0.94	0.528	1.59	<0.001
	0.76-1.15		1.25-2.03	
**Exposure hours to COVID-19 news and information on social media**				
0	Ref ^§^			
1	1.14	0.137	1.04	0.710
	0.96-1.35		0.85-1.26	
2-5	0.77	0.004	0.73	0.003
	0.64-0.92		0.59-0.90	
6+	0.62	<0.001	0.87	0.324
	0.49-0.78		0.67-1.14	
**Uses television to access COVID-19 news and information**				
No	Ref ^§^			
Yes	1.20	0.010	1.20	0.031
	1.04-1.39		1.02-1.41	
**Exposure frequency to COVID-19 news and information on television in the last week**				
Not once	Ref ^§^			
A few or some times	1.07	0.492	0.97	0.810
	0.88-1.30		0.78-1.21	
Frequently	0.84	0.112	0.92	0.502
	0.67-1.04		0.71-1.18	
**Exposure hours to COVID-19 news and information on television**				
0	Ref ^§^			
1	0.78	0.013	0.70	0.002
	0.64-0.95		0.56-0.88	
2	0.98	0.818	0.97	0.782
	0.79-1.21		0.76-1.23	
3+	0.83	0.094	0.84	0.162
	0.67-1.03		0.66-1.07	
**Uses radio to access COVID-19 news and information**				
No	Ref ^§^			
Yes	1.30	<0.001	1.03	0.724
	1.14-1.48		0.88-1.20	
**Exposure frequency to COVID-19 news and information on the radio in the last week**				
Not once	Ref ^§^			
A few or some times	1.01	0.852	0.77	0.003
	0.87-1.18		0.65-0.91	
Frequently	1.26	0.018	1.06	0.615
	1.04-1.53		0.85-1.32	
**Exposure hours to COVID-19 news and information on the radio**				
0	Ref ^§^			
1+	1.22	0.010	0.85	0.067
	1.05-1.42		0.71-1.01	

*p = p-value; ^†^Study population; ^‡^(95% CI) = 95% Confidence Interval; ^§^Ref = Reference category

For depression screening and in the independent regression of the blocks, the variables that presented statistically significant differences were as follows: Block 1, country of origin; Block 2, gender (male), age group, marital status (separated/divorced), race/skin color (non-white), number of people living in the same house, area of residence, maximum schooling level (others) and change in income during the COVID-19 pandemic (decrease in income); Block 3, use, exposure frequency (some times and frequently) and exposure hours (at least one) to COVID-19 news and information on social networks, use and exposure hours (one hour or more) to COVID-19 news and information on television, and use and exposure hours (at least one) to COVID-19 news and information on the radio.

In the final model (Table 5) and with Nagelkerke’s R²=0.169 and p-value<0.001 as per Hosmer-Lemeshow test, the following variables presented p-values<0.05: country of origin, gender (male), age group (at least 80 years old), race/skin color (non-white), maximum schooling level (from Elementary School or Basic Education upwards), change in income during the COVID-19 pandemic (decrease in income), use and exposure hours (at least one) to COVID-19 news and information on television, and exposure hours (at least one) to COVID-19 news and information on the radio.

**Table 5-:** Final model of screening/not screening for geriatric depression and p-values* corresponding to socioeconomic and demographic characteristics and exposure to COVID-19 news and information among older adults (n^†^ = 7,976). Peru, Brazil, Mexico, 2022

**Variables**	**Exposition (B) adjusted per** **block (95% CI)** ^‡^	p*	**Exposition (B) adjusted in the final model (95% CI)** ^‡^	p*
**Block 1 - Participants’ origin variable**				
**Country of origin**				
Peru	Ref ^§^			
Brazil	0.23 0.21-0.26	<0.001	0.23 0.19-0.26	<0.001
Mexico	0.49 0.39-0.63	<0.001	0.45 0.34-0.60	<0.001
**Block 2 - Socioeconomic and demographic variables**				
**Biological sex**				
Female	Ref ^§^			
Male	0.88 0.80-0.98	0.014	0.83 0.75-0.92	0.001
I prefer not to answer	1.29 0.49-3.41	0.610	2.07 0.79-5.44	0.138
**Age group (years)**				
60-64	Ref ^§^			
65-69	1.26 1.12-1.43	<0.001	1.17 1.04-1.33	0.012
70-74	1.26 1.09-1.46	0.002	1.09 0.94-1.27	0.241
75-79	1.29 1.09-1.51	0.003	1.15 0.97-1.37	0.110
80+	1.70 1.43-2.03	<0.001	1.43 1.18-1.72	<0.001
**Marital status**				
Single	Ref ^§^			
Married/Living together	0.98 0.83-1.15	0.775	1.08 0.91-1.28	0.405
Separated/Divorced	0.77 0.63-0.94	0.010	0.87 0.71-1.07	0.200
Widowed	1.02 0.85-1.22	0.864	1.05 0.86-1.26	0.647
**Race/Skin color**				
White	Ref ^§^			
Non-white	1.62 1.47-1.80	<0.001	0.86 0.76-0.98	0.022
**Number of people living in the same house**				
Lives alone	Ref ^§^			
One or two	1.19 1.01-1.41	0.043	1.05 0.88-1.25	0.583
Three or more	1.46 1.22-1.74	<0.001	0.96 0.80-1.15	0.655
**Own house**				
No	Ref ^§^			
Yes	0.93 0.82-1.05	0.259	0.93 0.81-1.05	0.246
**Area of residence**				
Urban	Ref ^§^			
Rural	1.46 1.26-1.69	<0.001	1.09 0.93-1.27	0.283
**Maximum schooling level**				
Did not study or Incomplete Basic Education	Ref ^§^			
Basic Education or Elementary School	0.91 0.75-1.10	0.337	0.76 0.62-0.93	0.007
High School	1.00 0.82-1.21	0.990	0.71 0.58-0.88	0.001
Complete Higher Education	1.12 0.92-1.36	0.246	0.71 0.57-0.88	0.002
Others	0.70 0.56-0.87	0.001	0.66 0.52-0.83	<0.001
**Change in income during the COVID-19 pandemic**				
No	Ref ^§^			
Yes, it increased	1.26 0.97-1.63	0.084	0.97 0.74-1.27	0.815
Yes, it decreased	1.66 150-1.83	<0.001	1.19 1.07-1.33	0.001
**Block 3 - Exposure to COVID-19 news and information variables**				
**Uses social media to access COVID-19 news and information**				
No	Ref ^§^			
Yes	1.15 1.01-1.31	0.037	1.13 0.99-1.30	0.078
**Exposure frequency to COVID-19 news and information on social media in the last week**				
Not once	Ref ^§^			
A few or some times	0.81 0.69-0.96	0.015	1.12 0.94-1.34	0.216
Frequently	0.68 0.55-0.84	<0.001	1.00 0.80-1.26	0.968
**Exposure hours to COVID-19 news and information on social media**				
0	Ref ^§^			
1	0.82 0.69-0.98	0.026	0.86 0.71-1.04	0.114
2-5	0.82 0.68-0.98	0.031	0.92 0.76-1.12	0.398
6*	0.76 0.60-0.95	0.017	1.08 0.85-1.38	0.529
**Uses television to access COVID-19 news and information**				
No	Ref ^§^			
Yes	0.79 0.68-0.91	0.001	0.86 0.73-1.00	0.047
**Exposure frequency to COVID-19 news and information on television in the last week**				
Not once	Ref ^§^			
A few or some times	1.01 0.83-1.23	0.940	0.91 0.74-1.12	0.362
Frequently	0.88 0.70-1.09	0.244	0.94 0.74-1.19	0.590
**Exposure hours to COVID-19 news and information on television**				
0	Ref ^§^			
1	1.44 1.18-1.76	<0.001	1.36 1.10-1.68	0.005
2	1.41 1.13-1.75	0.002	1.36 1.09-1.72	0.008
3+	1.59 1.28-1.97	<0.001	1.66 1.32-2.09	<0.001
**Uses radio to access COVID-19 news and information**				
No	Ref ^§^			
Yes	1.23 1.08-1.41	0.002	1.08 0.93-1.25	0.305
**Exposure frequency to COVID-19 news and information on the radio in the last week**				
Not once	Ref ^§^			
A few or some times	1.05 0.90-1.22	0.541	0.90 0.77-1.06	0.217
Frequently	1.06 0.87-1.29	0.539	0.98 0.80-1.21	0.868
**Exposure hours to COVID-19 news and information on the radio**				
0	Ref ^§^			
1+	1.49 1.28-1.74	<0.001	1.21 1.03-1.43	0.020

*p = p-value; ^†^Study population; ^‡^(95% CI) = 95% Confidence Interval; ^§^Ref = Reference category

## Discussion

This study investigated the association between exposure to COVID-19 news and information and the impact on older adults’ mental health in Peru, Brazil and Mexico. The prevalence of geriatric anxiety screening among the participants was 42.6%. It is worth noting that the prevalence was only higher for the outcome in the sample of older adults from Peru: 63.5%. For depressive symptoms, 59.4% of the sample indicated screening, while the frequency values were 74.1% and 58.6% in the samples of Peruvian and Mexican participants, respectively.

The findings are above the frequency found in a systematic review^(13)^ focused on Latin America, which estimates 35% (95% CI: 31%-38%) prevalence for anxiety and 35% (95% CI: 31%-39%) for depressive symptoms. When observing other world regions^(11)^, the prevalence ranges from 7.4% to 47.82% for anxiety symptoms and from 14.14% to 48.3% for depression. However, it is pointed out that the reviews do not have samples exclusively comprised by aged people and that the instruments used to screen symptoms differ from those applied in the study. Furthermore, the beginning of the data collection period coincided with the first highest peak of deaths due to COVID-19 recorded in the Americas, with emphasis on January 18^th^, 2021, which may have also contributed to the findings being different than those in the literature^(1)^.

Regarding media use to access COVID-19 news and information, 77.6% of the participants stated resorting to television, 46.6% used social media and 42.7% listened to the radio for this purpose. Only the sample of Brazilian aged people showed prevalence of social media use, at 58.8%. Only the sample of Peruvians reported listening to the radio, at 57.4%. The Mexican participants stood out for not using television, with 54.8%, and did not present any predominantly positive frequency for any of the three media.

It can be seen that none of the three countries agreed on any of the items. In a global journalism analysis at the beginning of 2023, Internet access in Peru, Brazil and Mexico was indicated at 87%, 83% and 67%, respectively; in turn, online access (including social networks) was the main source to access news, although a general decrease in this exposure was perceived. This reduction was also identified in traditional media such as television and printed newspapers^(23)^.

In Brazil, online news consumption (including social networks) has decreased from 90% to 79% in the last 10 years, with WhatsApp use standing out. Television is indicated by 51% of the participants. Similarly, Peru and Mexico present online news consumption (including social networks) at 80%, mainly through Facebook. They differ in relation to television, with consumption at 51% in Peru and at 42% in Mexico^(23)^.

The differences in findings among the countries are a driver to emphasize the context of these territories and understand the information-related behaviors of the sample population, which is exclusively made up of aged people. It is observed that the participants engaged with COVID-19 news and information even via digital media (social networks). However, the information quality and the aged people’s trust and literacy levels were not measured.

In a pre-pandemic study on access to digital media by older people in some Latin American countries (including Peru and Mexico), aged individuals made up less than 10% of the user population, with emphasis on use for information, followed by communication^(24)^. During the pandemic^(8)^, they point out that the inability to recognize fake news reached 79% of the population in Peru, above Mexico with 66% and Brazil, at 62%.

This is combined with low digital literacy^(25)^ and declining levels of public trust in the news among aged people, mainly due to the political situation and to their presidents’ behaviors, namely Brazil with 43% trust, followed by Mexico at 36% and Peru at 33%^(23)^.

In our sample, the bivariate association of media use for the anxiety screening outcome showed statistically significant differences for the use of social networks (X²=43.6 and p-value<0.001) and radio (X²=135.0 and p-value<0.001). However, only for radio was there 50% frequency for the outcome to occur or not. Regarding depression screening, for social networks (X²=29.0 and p-value<0.001), television (X²=3.83 and p-value<0.050) and radio (X²=138.0 and p-value<0.001), the frequency for the outcome was higher regardless of use.

The impact of digital social media on the perception of loneliness and social isolation among aged people was analyzed in an integrative review, which indicated that using these media has the potential to reduce such feelings, promoting greater interaction and a sense of belonging among older adults^(26)^.

These findings partially converged with the results of the current study, as we also identified a significant association between exposure to social media and changes in the participants’ mental health during the pandemic. However, unlike the results found in the integrative review, our study indicated that frequent exposure to social media was associated with higher prevalence of anxiety and depression symptoms, suggesting that the pandemic context may have exerted a negative influence on this relationship^(26)^.

Thus, the findings indicate that aged people are connected to COVID-19 information and can be screened for mental disorders (especially depression) but that, within the limits of the method and analyses, it is not possible to infer the direction of the reactions considering the context known to the participants.

In the final model for geriatric anxiety screening, with 31.18% variance explained by Nagelkerke’s R² and p-value<0.001 as per Hosmer-Lemeshow and controlled for socioeconomic factors, it is estimated that the aged people participating in Brazil had 0.10 (95% CI: 0.09-0.12 and p-value<0.001) times the chance of screening for anxiety and that those in Mexico presented 0.05 (95% CI: 0.03-0.08 and p-value<0.001) times the chance, in relation to the reference category: aged people in Peru.

Regarding exposure to COVID-19 news and information, all three media presented different significance values. Using social networks for this purpose presented 1.59 (95% CI: 1.25-2.03 and p-value<0.001) times the chance of screening for geriatric anxiety in relation to those who did not use it this way. Similarly, from 2 to 5 exposure hours on social networks had 0.73 (95% CI: 0.59-0.90 and p-value=0.003) times the chance of screening in relation to those that did not expose themselves at any moment.

Regarding television, its use for COVID-19 information represented 1.20 (95% CI: 1.02-1.41 and p-value=0.031) times the chance for the outcome in relation to those that did not resort to it. In the case of one hour, the chance for screening was 0.70 (95% CI: 0.56-0.88 and p-value=0.002) times higher in relation to those with no exposure hours with this intention. In the case of radio, only the “once or twice a week” frequency indicated 0.77 (95% CI: 0.65-0.91 and p-value=0.003) times the chance for the outcome for those who did not expose themselves to COVID-19 information.

In the second model, with outcome for depression and 16.89% explained variance by Nagelkerke’s R² and p-value<0.001 as per Hosmer-Lemeshow controlled by socioeconomic factors, the Brazilians presented 0.23 (95% CI: 0.19-0.26 and p-value<0.001) times the chance of screening and the Mexicans reached 0.45 (95% CI: 0.34-0.60 and p-value<0.001) times the chance, in relation to the aged Peruvians.

Significance for the outcome was only verified in television and radio. At least three exposure hours to COVID-19 news and information on television was 1.66 (95% CI: 1.32-2.09 and p-value<0.001) times higher than among those that did not spend any time on this medium. Exposure time was also relevant in relation to radio, with at least one hour being 1.21 (95% CI: 1.03-1.43 and p-value=0.020) times higher than in those that did not resort to this option.

These results reassert the association between media consumption (digital or otherwise) of COVID-19 information and the possibility of screening for the mental disorders analyzed in the aged participants. In none of the associated variables did absence or low exposure to any of the media indicate a higher probability for the outcomes.

In a systematic review^(27)^, social media use is one of the causes of the infodemic, with psychological issues standing out among the impacts. The opportune environment for misinformation flow (in various ways) was social networks and it inevitably exceeded its limits. In the “infodemic vicious circle”^(27)^, the impact on mental health amplifies the infodemic machinery that results in worsening of disorders.

Inequality of access to good quality information, updated and consistent with the needs of individuals and communities, is discussed in the infodemic management movement. In terms of the potential harms to health, emotional states resulting from information overload may not begin with the information itself but involve previously existing states, with the capability of influencing people’s behaviors^(6)^.

Limited by the objective of the article, it is not possible to encompass all the nuances inherent to the complexity of information and health; however, it is consistent with the discussion^(28)^ that information overload is not an acute process but a structural one, which is part of the social determination of health. Information inequality, the infodemic, ageism, media monopolies and the asylum health model operate in the same universe marked by life commercialization. Breaking these structures foresees not only antagonism but the construction of a societal project that also includes guaranteeing the right to good quality, free, safe and accessible information.

A limitation of this study is the effort made to use the same screening instruments for geriatric anxiety and depression in all three countries, which may have influenced the prevalence values. Furthermore, as is typical of cross-sectional observational studies, it is not possible to determine whether the chronological relationship between data collection and the COVID-19 pandemic would alter the findings. Furthermore, it is known that selection and information biases are possible in data collection via web-based surveys with non-probability sampling, restricting generalization of the findings.

This study represents a significant contribution to scientific advancement by demonstrating cross-national differences in the prevalence of geriatric anxiety and depression associated with media consumption during the pandemic among older adults in Peru, Brazil and Mexico. The findings are relevant to the Gerontology Nursing practice in these countries, as they guide specific strategies for promoting mental health and health literacy adapted to local realities.

## Conclusion

There was an association between frequent exposure to media and higher prevalence of geriatric anxiety and depression in the aged individuals under study. When comparing all three countries, the significant difference in prevalence of the outcomes was small. These results reinforce the need for specific strategies in managing the infodemic to protect the mental health of aged people living in Latin America.

Given the study limitations, the aged people in the Latin American countries analyzed are in consonance with the literature, which indicates that this population segment is present in digital media and adopts them (focusing on social networks in this study). In addition, their mental health, which extends to their relationships with information, corroborates the findings on the demand and need for care and targeted public policies. In view of this, it is emphasized that the intention is not to delimit and reinforce biomedical stereotypes of the experience and relationship of individuals with mental disorders; as possible symptoms alone do not determine people’s behaviors and choices.

The research provides an opportunity to add to the knowledge produced and focused on Latin America, with current articulation linked to the COVID-19 pandemic and future implications and articulation to population aging and management of the infodemic as a Public Health policy. The expectation is to contribute to research studies that add to the right to information and healthy aging, investing in prevention and health promotion through education (health literacy) and participation (listening to health demands and needs) to devise and/or strengthen intersectoral public policies that take into consideration the power of Latin American peoples.
