# Supplementary material for: Cross-national prevalence of mental disorders in older adults exposed to COVID-19 information
Source: Rev Lat Am Enfermagem. 2025 Aug 18;33:e4632. doi: 10.1590/1518-8345.7580.4632 (PMC12364455; doi:10.1590/1518-8345.7580.4632)
Supplement: Translation

# Translation: Prevalência transnacional de distúrbios psíquicos em pessoas idosas expostas a informações sobre a COVID-19*

**DOI:** 10.1590/1518-8345.7580.4633

**Authors:** Daniele Knopp Ribeiro, Fábio da Costa Carbogim, Patricia Rodrigues Braz, Sofia Sabina Lavado-Huarcaya, Aracely Díaz-Oviedo, Alexandre Favero Bulgarelli, Rosimere Ferreira Santana, Ione Carvalho Pinto, Fabiana Costa Machado Zacharias, Ricardo Bezerra Cavalcante

**Affiliations:** 1 Universidade Federal de Juiz de Fora, Juiz de Fora, MG, Brasil.; 2 Bolsista da Coordenação de Aperfeiçoamento de Pessoal de Nível Superior (CAPES), Brasil.; 3 Universidad Señor de Sipán, Escuela de Postgrado, Pimentel, Lambayeque, Peru.; 4 Universidad Autónoma de San Luís Potosí, Facultad de Enfermería y Nutrición, San Luis Potosí, SLP, Mexico.; 5 Universidade Federal do Rio Grande do Sul, Porto Alegre, RS, Brasil.; 6 Universidade Federal Fluminense, Faculdade Enfermagem, Niterói, RJ, Brasil.; 7 Universidade de São Paulo, Escola de Enfermagem de Ribeirão Preto, Centro Colaborador da OPAS/OMS para o Desenvolvimento da Pesquisa em Enfermagem, Ribeirão Preto, SP, Brasil.

**Keywords:** Infodemia, Saúde Mental, Idoso, América Latina, Covid-19, Estudos Transversais

## Abstract

verificar a associação entre exposição a notícias e informações sobre COVID-19, por redes sociais, televisão e rádio, e o rastreio para ansiedade e depressão geriátrica comparando Peru, Brasil e México.

delineamento transversal, por *web-based survey*, em amostra não probabilística, com escalas validadas para rastreio de ansiedade e depressão geriátrica, e análise dos dados por regressão logística binária hierárquica.

dos 7.976 participantes, prevaleceu o sexo feminino (n = 4.937, 61,9%), cor/raça não branca (n = 4.724, 59,2%) e na faixa etária de 60 a 64 anos (n = 2.584, 32,4%). O acesso às notícias e informações sobre COVID-19 ocorreu pela televisão (n = 6.187, 77,6%), em poucas ou algumas vezes na semana (n = 4.322, 54,2%) por três horas ou mais (n = 2.596, 32,5%). Nos modelos finais, para ansiedade ou depressão, as diferenças significativas (p-valor < 0,001) para os aspectos uso, frequência e horas de exposição alteraram a depender da mídia. A prevalência para os desfechos, em comparação aos três países, foi pequena.

a exposição frequente às mídias foi associada a uma maior prevalência de ansiedade e depressão geriátrica, embora a diferença entre os países estudados tenha sido pequena.

## Destaques

**(1)** As variáveis associadas aos desfechos indicam o consumo de notícias e informações. **(2)** A frequência e o tempo de exposição às mídias importam para o rastreio. **(3)** A prevalência para o desfecho em comparação aos três países foi pequena. **(4)** O rastreio para ansiedade foi de 42,6% e para depressão 59,4%. **(5)** O consumo midiático foi de 77,6% televisão, 46,6% redes sociais e 42,7% rádio.

## Introdução

Em 30 de janeiro de 2020 e posteriormente em 11 de março do mesmo ano, o risco à saúde pública e a capacidade de disseminação do coronavírus SARS-CoV-2, responsável pela doença COVID-19, foram reconhecidos. Cerca de quatro anos após, mais de 772 milhões de casos e aproximadamente 7 milhões de mortes foram notificados mundialmente, com destaque para as regiões da Europa e das Américas na prevalência de casos e mortes notificadas, respectivamente^(1)^.

A saúde da população da América Latina e do Caribe foi a mais impactada do mundo na pandemia por COVID-19. Mesmo com os diferentes arranjos dos sistemas de saúde e diversidade social e política, grupos historicamente vulnerabilizados, a citar alguns como as mulheres, não brancos, pessoas em situação de pobreza e/ou de rua, estudantes e pessoas idosas, vivenciaram diferentemente a pandemia e sofreram com o agravamento das desigualdades históricas da região^(2-3)^.

A complexidade da experiência pandêmica foi acrescida pelo excesso e velocidade de informações produzidas e disseminadas nos meios tradicionais de comunicação em massa, sendo amplificada, ainda, pelas redes sociais. Nesse contexto, é moroso reconhecer a origem, intenção e qualidade das informações, e a esse fenômeno, denomina-se infodemia^(4)^. Podendo resultar em alterações na percepção de risco, sentimentos e sensações de confusão e desorientação, hesitação, paralisação, negação, desconfiança, dentre outros, que se relacionam às experiências prévias dos indivíduos e com pessoas de confiança, o sistema de saúde e o governo da região^(5-6)^.

Em menos de um ano da reconhecida pandemia, a incipiência na estrutura e coordenação dos governos latino-americanos com a gestão da infodemia foi relatada^(7)^, com base na análise dos *sites* dos ministérios da saúde de 10 países. Em paralelo^(8)^, em estudo ecológico descritivo, destacam que em seis países latino-americanos investigados, os locais com maior dificuldade em reconhecer notícias falsas e a centralidade das redes sociais como meio de informação, coincidiram com as mais altas taxas de mortalidade por COVID-19 naquele período.

Além disso, na mídia jornalística do Brasil e do Chile em 2020/2021, discursos de “já viveram suas vidas”, culpabilização pela gravidade da pandemia e homogeneidade das mortes puderam ser encontrados ao referenciar as pessoas idosas. Ainda que também se reconheça o concebível agravamento na saúde mental pós-pandemia, decorrente da ridicularização, abandono, dependência e controle a que essa população foi submetida^(9)^.

O agravamento da vulnerabilização de pessoas idosas perpassa a histórica condição socioeconômica-demográfica de iniquidades, associado ao sabido aumento do envelhecimento populacional, que no período pandêmico por COVID-19, é escancarado na taxa de mortalidade proporcional ao aumento da idade^(1-2)^. A necessidade de investigação, cuidado e fortalecimento de políticas públicas integra o movimento pela Década do Envelhecimento Saudável 2021─2030, que nas Américas é coordenada pela Organização Panamericana da Saúde (OPS)^(10)^.

Em meio à incipiência de estudos com a população exclusivamente de pessoas idosas e/ou das implicações na saúde mental decorrente da infodemia em países latino-americanos, como no Peru, Brasil e México, justifica-se o presente estudo. Embora se considere que sintomas complexos como ansiedade, depressão e insônia, durante a pandemia, estejam relatados em revisões sistemáticas com a população adulta em diferentes regiões do mundo, ao associar com as mídias, tem-se o foco majoritariamente para mídias sociais^(11-13)^.

Assim, objetiva-se verificar a associação entre exposição a notícias e informações sobre COVID-19 por redes sociais, televisão e rádio, e o rastreio para ansiedade e depressão geriátrica, comparando pessoas idosas do Peru, Brasil e México.

## Método

### Delineamento do estudo

Estudo transversal, realizado com pessoas idosas maiores de 60 anos, no período de julho de 2020 a junho de 2022, no Peru, Brasil e México. Derivado da parte 1 da pesquisa “Infodemia de COVID-19 e suas repercussões sobre a saúde mental de idosos durante e pós-pandemia: estudo multicêntrico Brasil, Peru, Colômbia, México e Portugal”, que objetiva “analisar a relação entre a infodemia sobre a COVID-19 e as repercussões na saúde mental de pessoas idosas”.

### Aspectos éticos

O estudo foi aprovado pelo Comitê de Ética e Pesquisa em Seres Humanos no Brasil (CAAE: 31932620.1.1001.5147, sob o parecer n.º 4.134.050), Peru e México (Universidade Autônoma de San Luis Potosí, sob registro CONBIOÉTICA-24-CEI-002-20230925).

### Critérios de seleção

Foram incluídas pessoas idosas maiores de 60 anos, com cognitivo preservado, acesso a e-mail e/ou redes sociais e/ou telefone. Foram excluídas pessoas idosas que não possuíam condições de responder aos questionamentos com autonomia, conforme autorrelato da pessoa contatada.

### Cenários

Os participantes residem em uma das seguintes cidades: Brasil - Brasília, Divinópolis, Juiz de Fora, Porto Alegre, Rio de Janeiro, Ribeirão Preto, São Paulo e Viçosa; México ― Cuidad Valles, Matehuala, Rioverde, Salinas de Hidalgo, San Luis Potosí, Soledas de Graciano Sánchez e Tamazunchale; Peru ― Arequipa, Cerro de Pasco, Chiclayo, Huánuco, Iquitos, Lima, Puno-Juliaca, Tacna, Tarapoto, Tumbes e Trujillo.

### Coleta de dados

A coleta de dados ocorreu por *web-based survey*, em amostra não probabilística, divulgada por *e-mail*, redes sociais (*WhatsApp*, *Facebook* e *Instagram*) e difundidas por sociedades científicas de geriatria e gerontologia, instituições de assistência à saúde, associações de aposentados e centros de pesquisa. Foram realizadas 15 entrevistas-piloto em cada país para adequação do idioma às questões.

O *link* para acesso ao questionário direcionou para o Termo de Consentimento Livre e Esclarecido (TCLE). Somente os participantes que sinalizaram a concordância, tiveram acesso às questões, todas sinalizadas como obrigatórias. Todos receberam uma cópia do TCLE assinado pelo pesquisador por e-mail ou redes sociais. As pessoas idosas também foram contatadas por telefone, como convite a participarem da pesquisa, no momento da ligação ou em remarcação, com leitura do TCLE e sinalização de concordância da gravação da ligação. Posteriormente, também foi enviado o TCLE assinado pelo pesquisador por *e-mail* ou redes sociais.

### Instrumentos

O questionário foi composto pelos segmentos de identificação, perfil sociodemográfico, exposição a notícias e informações relacionadas à COVID-19^(14-15)^, impacto de sinais e sintomas sobre alterações psicopatológicas ao contato com as informações sobre COVID-19 (pesquisa em desenvolvimento), Escala de Estresse Percebido, Inventário de Ansiedade Geriátrica (GAI)^(16)^ e a Escala de Depressão Geriátrica (GDS-15)^(17)^. O presente estudo analisa os segmentos perfil sociodemográfico, exposição a notícias e informações relacionadas à COVID-19, GAI e GDS-15^(16-17)^.

Para os dados coletados no Brasil, a GAI^(18)^, validada com pessoas idosas do país, é composta por 20 itens autorrespondidos em questões binárias (concordo/discordo) e ponto de corte < 13 não caso e ≥ 13 caso. A GDS-15^(19)^, igualmente validada, contém 15 perguntas das quais 10 pontuam se respondidas positivamente e as demais respondidas negativamente, com ponto de corte < 6 para não caso e ≥ 6 para caso.

Os dados coletados no México seguem a GAI^(20)^, versão validada em espanhol com pessoas idosas de Madri, na ausência de obtenção de estudo de validação no país. O inventário é composto por 20 itens dicotômicos (concordo/discordo), com indicação de ponto de corte para ≥ 11 para caso e < 11 para não caso. A GDS-15, validada com pessoas idosas mexicanas^(21)^, é composta por 15 questões, com opções de sim ou não, em que 10 questões pontuam se respondidas positivamente e as demais respondidas negativamente. Os autores não indicam ponto de corte, comparou-se com escores altos (10 ou mais sintomas) e escores baixos (menos de cinco sintomas) para depressão. Entendendo, caso ≥ 5 e não caso < 5.

Aos dados do Peru, seguiu-se a GAI^(20)^, versão validada em espanhol com pessoas idosas de Madri, na ausência de obtenção de estudo de validação no país. O inventário é composto por 20 itens dicotômicos (concordo/discordo), com indicação de ponto de corte para ≥ 11 para caso e < 11 para não caso. Na avaliação da sintomatologia para depressão, utilizou-se o referencial com validação para pessoas idosas colombianas, composto por 15 itens dicotômicos em sim ou não, em que 10 questões pontuam se respondidas positivamente e as demais respondidas negativamente. O ponto de corte proposto foi de ≥ 5 para sugestão de depressão^(22)^.

As variáveis independentes relativas aos desfechos foram organizadas nos seguintes blocos hierarquizados do modelo de análise:

Bloco 1- Variável de origem dos participantes: Peru, Brasil e México.

Bloco 2 - Variáveis socioeconômicas e demográficas: sexo; faixa etária; estado civil; raça/cor; mora com; situação da residência; área de residência; educação máxima; alteração da renda devido à pandemia de COVID-19.

Bloco 3 - Variáveis de exposição a notícias e informações sobre COVID-19: tipo de mídia; frequência de exposição na última semana; horas de exposição.

### Tratamento e análise dos dados

Na análise dos dados coletados, os participantes receberam um código numérico para manutenção do sigilo, a tabulação foi desenvolvida no Planilhas *Google* e enviada para o *software* IBM *Statistical Package for the Social Sciences* (SPSS) versão 23.0 para *Windows*. Todas as variáveis foram submetidas a análise descritiva, calculadas distribuição de frequências absoluta (n) e relativa (%). Para análise das associações, o teste do qui-quadrado (X^2^) de independência de Pearson foi utilizado na análise bivariada de cada bloco para as variáveis independentes categóricas nominais, e o teste U de Mann-Whitney para as variáveis independentes ordinais.

Seguido da análise de regressão logística binária hierárquica, na qual, inicialmente, todas as variáveis nos respectivos blocos compuseram análises separadamente. Para comparação, conforme o bloco de origem, as variáveis foram introduzidas no modelo multivariado final. Para todos os testes, considerou-se nível de significância de 5% e intervalo de confiança de 95%.

## Resultados

Dos 7.976 participantes, 4.377 (54,9%) procederam do Peru, 3.307 (41,5%) do Brasil e 292 (3,7%) do México. Desta amostra, 4.937 (61,9%) declararam ser do sexo feminino e 4.724 (59,2%) referiram ser de cor/raça não branca, incluindo pretos, pardos, amarelos e ascendência indígena. A faixa etária mais frequente se concentrou entre 60 e 64 anos, com 2.584 (32,4%) pessoas idosas. Quanto ao estado civil, 4.642 (58,2%) pessoas idosas declararam ser casadas ou estar morando com o(a) parceiro(a).

Em relação à moradia, 6.514 (81,7%) informaram possuir residência própria, 6.830 (85,6%) residem em área urbana e 3.744 (46,9%) residem com três pessoas ou mais. Sobre a educação máxima indicada, o ensino superior completo com 2.120 (26,6%) pessoas idosas foi o mais frequente. Quanto à influência da pandemia da COVID-19 na renda, 4.112 (51,6%) dos participantes consideraram que não houve alteração na renda.

Sobre informações da COVID-19 em redes sociais, 4.259 (53,4%) participantes referiram não utilizar as redes sociais, sendo que 3.261 (40,9%) não declararam nenhuma exposição na semana anterior à pesquisa.

Para 6.187 (77,6%) participantes, a televisão (TV) foi utilizada para acessar notícias e informações sobre a COVID-19, contudo, 4.322 (54,2%) referiram assistir poucas vezes ou algumas vezes à TV na semana anterior à participação na pesquisa. Para 2.596 (32,5%) idosos, a utilização da TV ficou na faixa de três horas ou mais por semana.

Quanto à utilização do rádio, 4.568 (57,3%) referiram não ter acessado notícias ou informação sobre a COVID-19 nenhuma vez por esse meio na semana anterior à participação na pesquisa. Os dados tabulados pelos países podem ser observados na Tabela 1 a seguir:

**Tabela 1-:** Frequências segundo características socioeconômicas e demográficas, e exposição a notícias e informações sobre COVID-19 de pessoas idosas (n* = 7.976). Peru, Brasil, México, 2022

**Variáveis**	**Países**							
	**Peru** **Frequência** **(n = 4.377)** ^†^		**Brasil** **Frequência** **(n = 3.307)** ^‡^		**México** **Frequência** **(n = 292)** ^§^		**Total** **Frequência** (n = 7.976)*	
	**N** ^||^	**%** ^¶^	**N** ^||^	**%** ^¶^	**N** ^||^	**%** ^¶^	**N** ^||^	**%** ^¶^
**Bloco 2 - Variáveis socioeconômicas e demográficas**								
**Sexo biológico**								
Feminino	2.452	56,0	2.250	68,0	239	81,8	4.941	61,9
Masculino	1.925	44,0	1.039	31,4	53	18,2	3.017	37,8
Prefiro não declarar	0	0,0	18	0,5	0	0,0	18	0,2
**Faixa etária (anos)**								
60-64	1.241	28,4	1.285	38,9	61	20,9	2.587	32,4
65-69	1.284	29,3	921	27,9	86	29,5	2.291	28,7
70-74	705	16,1	503	15,2	60	20,5	1.268	15,9
75-79	547	12,5	334	10,1	43	14,7	924	11,6
80 ou acima	600	13,7	264	8,0	42	14,4	906	11,4
**Estado civil**								
Solteiro(a)	423	9,7	365	11,0	36	12,3	824	10,3
Casado(a)/morando junto	2.670	61,0	1.835	55,5	137	46,9	4.642	58,2
Separado(a)/desquitado(a)	322	7,4	509	15,4	23	7,9	854	10,7
Viúvo(a)	962	22,0	598	18,1	96	32,9	1.656	20,8
**Raça/cor**								
Branco	607	13,9	2.364	71,5	281	96,2	3.252	40,8
Não branco	3.770	86,1	943	28,5	11	3,8	4.724	59,2
**Quantidade de moradores no domicílio**								
Mora sozinho	189	4,3	587	17,8	33	11,3	809	10,1
Uma ou duas	1.412	32,3	1.886	57,0	125	42,8	3.423	42,9
Três ou mais	2.776	63,4	834	25,2	134	45,9	3.744	46,9
**Residência própria**								
Não	872	19,9	551	16,7	39	13,4	1462	18,3
Sim	3.505	80,1	2.756	83,3	253	86,6	6.514	81,7
Área de residência								
Urbana	3.380	77,2	3.160	95,6	290	99,3	6.830	85,6
Rural	997	22,8	147	4,4	2	0,7	1.146	14,4
**Educação m** áxima								
Não estudou ou não concluiu o ensino básico	355	8,1	295	8,9	25	8,6	675	8,5
Ensino básico ou fundamental	1.133	25,9	713	21,6	116	39,7	1.962	24,6
Ensino médio	1.305	29,8	718	21,7	56	19,2	2.079	26,1
Ensino superior completo	1.446	33,0	645	19,5	29	9,9	2.120	26,6
Outros	138	3,2	936	28,3	66	22,6	1.140	14,3
**Alteração da renda durante a pandemia por COVID-19**								
Não	1.536	35,1	2.437	73,8	139	47,6	4.112	51,6
Sim, a minha renda aumentou	172	3,9	80	2,4	15	5,1	267	3,3
Sim, a minha renda diminuiu	2.669	61,0	787	23,8	138	47,3	3.594	45,1
**Bloco 3 - Variáveis de exposição a notícias e informações sobre COVID-19**								
Utiliza redes sociais para acessar notícias e informações sobre COVID-19* *								
Não	2.719	62,1	1.361	41,2	179	61,3	4.259	53,4
Sim	1.658	37,9	1.943	58,8	113	38,7	3.714	46,6
**Frequência de exposição na última semana a notícias e informações sobre COVID-19 em redes sociais**								
Nenhuma vez	2.280	52,1	822	24,9	159	54,5	3.261	40,9
Poucas vezes e algumas vezes	1.571	35,9	1.464	44,3	102	34,9	3.137	39,3
Frequentemente	526	12,0	1.021	30,9	31	10,6	1.578	19,8
Horas de exposição a notícias e informações sobre COVID-19 em redes sociais**								
0 h	2.097	47,9	848	25,7	182	62,3	3.127	39,2
1 h	1.080	24,7	811	24,6	49	16,8	1.940	24,3
2 h a 5 h	1.004	22,9	1.084	32,8	58	19,9	2.146	26,9
6 h ou acima	196	4,5	560	17,0	3	1,0	759	9,5
Utiliza televisão para acessar notícias e informações sobre COVID-19**								
Não	1.002	22,9	624	18,9	160	54,8	1.786	22,4
Sim	3.375	77,1	2.680	81,1	132	45,2	6.187	77,6
**Frequência de exposição na última semana a notícias e informações sobre COVID-19 pela televisão**								
Nenhuma vez	601	13,7	394	11,9	41	14,0	1.036	13,0
Poucas vezes e algumas vezes	2.669	61,0	1.440	43,5	213	72,9	4.322	54,2
Frequentemente	1.107	25,3	1.473	44,5	38	13,0	2.618	32,8
Horas de exposição a notícias e informações sobre COVID-19 pela televisão**								
0 h	597	13,6	431	13,1	53	18,2	1.081	13,6
1 h	1.595	36,4	884	26,8	111	38,0	2.590	32,5
2 h	947	21,6	686	20,8	71	24,3	1.704	21,4
3 h ou mais	1.238	28,3	1.301	39,4	57	19,5	2.596	32,5
Utiliza o rádio para acessar notícias e informações sobre COVID-19**								
Não	1.866	42,6	2.429	73,5	273	93,5	4.568	57,3
Sim	2.511	57,4	876	26,5	19	6,5	3.406	42,7
**Frequência de exposição na última semana a notícias e informações sobre COVID-19 pelo rádio**								
Nenhuma vez	1.317	30,1	1.956	59,1	211	72,3	3.484	43,7
Poucas vezes e algumas vezes	2.333	53,3	956	28,9	65	22,3	3.354	42,1
Frequentemente	727	16,6	395	11,9	16	5,5	1.138	14,3
Horas de exposição a notícias e informações sobre COVID-19 pelo rádio**								
0 h	1.330	30,4	2.083	63,0	223	76,4	3.636	45,6
1 h e mais	3.047	69,6	1.223	37,0	69	23,6	4.339	54,4

*População do estudo; ^†^Amostra total do Peru; **‡**Amostra total do Brasil; ^§^Amostra total do México; ^||^N = Distribuição dos participantes em cada resposta; ^¶^% = Porcentagem em relação ao total de respostas na coluna referente a cada variável; **Total de respondentes menor que a população total do estudo (7.976)

Das 7.976 pessoas idosas participantes, 3.395 (42,6%) alcançaram o rastreio para ansiedade geriátrica, enquanto 4.581 (57,4%) não alcançaram. Na análise bivariada das variáveis socioeconômicas e demográficas (bloco 2), os seguintes itens obtiveram diferenças significativas, sendo p-valor < 0,001: sexo biológico, faixa etária, país de origem, estado civil, raça/cor, quantidade de moradores no domicílio, área de residência e alteração da renda durante a pandemia por COVID-19. Ademais, o item educação máxima apresentou p-valor = 0,032 (Tabela 2 e Tabela 3).

Na associação da variável de rastreio/não rastreio para ansiedade com as variáveis de exposição a notícias e informações (bloco 3), observaram-se diferenças significativas nos itens: uso, frequência e horas de exposição pelas redes sociais para acesso a notícias e informações sobre COVID-19 com p-valor < 0,001. Para o uso, frequência e horas de exposição pelo rádio para acesso a notícias e informações sobre COVID-19 com p-valor < 0,001. Sobre a TV, somente as variáveis frequência e horas de exposição foram significativas, com p-valor < 0,001 e p-valor = 0,012, respectivamente.

Sobre o rastreio para depressão geriátrica, 4.734 (59,4%) indicaram o rastreio, à medida que 3.242 (40,6%) não obtiveram. As seguintes variáveis socioeconômicas e demográficas (bloco 1 e bloco 3) foram significativas na análise bivariada: faixa etária, país de origem, estado civil, raça/cor, quantidade de moradores no domicílio, área de residência, educação máxima e alteração da renda durante a pandemia por COVID-19, com p-valor < 0,001 cada item. Somente o item residência própria, dentre as diferenças significativas, atingiu p-valor = 0,008.

Da análise de associação bivariada entre o rastreio/não rastreio para depressão geriátrica e as variáveis de exposição a notícias e informações (bloco 3), os seguintes itens foram estatisticamente significativos: uso, frequência e horas de exposição pelas redes sociais para acesso a notícias e informações sobre COVID-19 com p-valor < 0,001 cada item. Para o uso, frequência e horas de exposição pelo rádio para acesso a notícias e informações sobre COVID-19, com p-valor < 0,001, respectivamente. Em relação à TV, somente o item frequência foi significativo, com p-valor < 0,001.

**Tabela 2-:** Rastreamento ou não para ansiedade e depressão geriátrica e valor de p* das características socioeconômicas e demográficas, e exposição a notícias e informações sobre COVID-19 de pessoas idosas (n^†^ = 7.976). Peru, Brasil, México, 2022

Variáveis	Rastreamento para ansiedade geriátrica				p*	Rastreamento para depressão geriátrica				p*
	Não		Sim			Não		Sim		
	N ^‡^	% ^§^	N ^‡^	% ^§^		N ^‡^	% ^§^	N ^‡^	% ^§^	
**Bloco 1 - Variável de origem dos participantes e Bloco 2 - Variáveis socioeconômicas e demográficas**										
**Sexo biológico**										
Feminino	2.985	65,2	1.956	57,6	<0,001	2.036	62,8	2.905	61,4	0,434
Masculino	1.584	34,6	1.433	42,2		1.199	37,0	1.818	38,4	
Prefiro não declarar	12	0,3	6	0,2		7	0,2	11	0,2	
**País**										
Peru	1.599	34,9	2.778	81,8	<0,001	1.134	35,0	3.243	68,5	<0,001
Brasil	2.712	59,2	595	17,5		1.987	61,3	1.320	27,9	
México	270	5,9	22	0,6		121	3,7	171	3,6	
**Estado civil**										
Solteiro	434	9,5	390	11,5	<0,001	352	10,9	472	10,0	<0,001
Casado/Morando junto	2.662	58,1	1.980	58,3		1.842	56,8	2.800	59,1	
Separado(a)/Desquitado(a)	552	12,0	302	8,9		432	13,3	422	8,9	
Viúvo	933	20,4	723	21,3		616	19,0	1.040	22,0	
**Raça/cor**										
Branco	2.492	54,4	760	22,4	<0,001	1.674	51,6	1.578	33,3	<0,001
Não branco	2.089	45,6	2.635	77,6		1.568	48,4	3.156	66,7	
**Residência própria**										
Não	828	18,1	634	18,7	0,501	549	16,9	913	19,3	0,008
Sim	3.753	81,9	2.761	81,3		2.693	83,1	3.821	80,7	
**Área de residência**										
Zona urbana	4.050	88,4	2.780	81,9	<0,001	2.920	90,1	3.910	82,6	<0,001
Zona rural	531	11,6	615	18,1		322	9,9	824	17,4	
**Bloco 3 - Variáveis de exposição a notícias e informações sobre COVID-19**										
**Utiliza redes sociais para acessar notícias e informações sobre COVID-19**										
Não	2.300	50,2	1.959	57,7	<0,001	1.613	49,8	2.646	55,9	<0,001
Sim	2.278	49,8	1.436	42,3		1.627	50,2	2.087	44,1	
**Utiliza televisão para acessar notícias e informações sobre COVID-19**										
Não	1.060	23,2	726	21,4	0,061	690	21,3	1.096	23,2	0,052
Sim	3.518	76,8	2.669	78,6		2.550	78,7	3.637	76,8	
**Utiliza rádio para acessar notícias e informações sobre COVID-19**										
Não	2.877	62,8	1.691	49,8	<0,001	2.111	65,2	2.457	51,9	<0,001
Sim	1.702	37,2	1.704	50,2		1.129	34,8	2.277	48,1	

*p = Qui-quadrado de Pearson; ^†^População do estudo; ^‡^N = Distribuição dos participantes em cada resposta; ^§^% = Porcentagem em relação ao total de respostas na coluna referente a cada variável

**Tabela 3-:** Rastreamento ou não para ansiedade e depressão geriátrica e valor de p* das características socioeconômicas e demográficas, e a exposição a notícias e informações sobre COVID-19 de pessoas idosas (n^†^= 7.976). Peru, Brasil, México, 2022

Variáveis	Rastreamento para ansiedade geriátrica				p*	Rastreamento para depressão geriátrica				p*
	Não		Sim			Não		Sim		
	N ^‡^	% ^§^	N ^‡^	% ^§^		N ^‡^	% ^§^	N ^‡^	% ^§^	
**Bloco 1 - Variável de origem dos participantes e Bloco 2 - Variáveis socioeconômicas e demográficas**										
**Faixa etária (anos)**										
60 – 64	1.570	34,3	1.017	30,0	<0,001	1.191	36,7	1.396	29,5	<0,001
65 – 69	1.303	28,4	988	29,1		889	27,4	1.402	29,6	
70 – 74	730	15,9	538	15,8		514	15,9	754	15,9	
75 – 79	507	11,1	417	12,3		358	11,0	566	12,0	
80 ou acima	471	10,3	435	12,8		290	8,9	616	13,0	
Quantidade de moradores no domicílio										
Mora sozinho	569	12,4	240	7,1	<0,001	427	13,2	382	8,1	<0,001
Uma ou duas pessoas	2.155	47,0	1.268	37,3		1.520	46,9	1.903	40,2	
Três ou mais pessoas	1.857	40,5	1.887	55,6		1.295	39,9	2.449	51,7	
**Educação máxima**										
Não estudou ou não concluiu o ensino básico	403	8,8	272	8,0	0,032	234	7,2	441	9,3	<0,001
Ensino básico ou fundamental	1.163	25,4	799	23,5		751	23,2	1.211	25,6	
Ensino médio	1.135	24,8	944	27,8		807	24,9	1.272	26,9	
Ensino superior completo	1.029	22,5	1.091	32,1		784	24,2	1.336	28,2	
Outros	851	18,6	289	8,5		666	20,5	474	10,0	
**Alteração da renda durante a pandemia por COVID-19**										
Não	2.564	56,0	1.548	45,6	<0,001	1.977	61,0	2.135	45,1	<0,001
Sim, a minha renda aumentou	149	3,3	118	3,5		105	3,2	162	3,4	
Sim, a minha renda diminuiu	1.866	40,8	1.728	50,9		1.158	35,7	2.436	51,5	
**Bloco 3 - Variáveis de exposição a notícias e informações sobre COVID-19**										
**Frequência de exposição na última semana a notícias e informações sobre COVID-19 em redes sociais**										
Nenhuma vez	1.686	36,8	1.575	46,4	<0,001	1.146	35,3	2.115	44,7	<0,001
Poucas vezes e algumas vezes	1.913	41,8	1.224	36,1		1.325	40,9	1.812	38,3	
Frequentemente	982	21,4	596	17,6		771	23,8	807	17,0	
**Horas de exposição a notícias e informações sobre COVID-19 em redes sociais**										
0 h	1.635	35,7	1.492	43,9	<0,001	1.118	34,5	2.009	42,4	<0,001
1 h	1.059	23,1	881	25,9		836	25,8	1.104	23,3	
2 h a 5 h	1.368	29,9	778	22,9		930	28,7	1.216	25,7	
6 h ou mais	515	11,3	244	7,2		355	11,0	404	8,	
Frequência de exposição na última semana a notícias e informações sobre COVID-19 pela televisão										
Nenhuma vez	572	12,5	464	13,7	<0,001	421	13,0	615	13,0	<0,001
Poucas vezes e algumas vezes	2.414	52,7	1.908	56,2		1.662	51,3	2.660	56,2	
Frequentemente	1.595	34,8	1023	30,1		1.159	35,7	1.459	30,8	
Horas de exposição a notícias e informações sobre COVID-19 pela televisão										
0 h	590	12,9	491	14,5	0,012	479	14,8	602	12,7	0,075
1 h	1.501	32,8	1.089	32,1		1.028	31,7	1.562	33,0	
2 h	924	20,2	780	23,0		708	21,9	996	21,0	
3 h ou mais	1.561	34,1	1.035	30,5		1.024	31,6	1.572	33,2	
Frequência de exposição na última semana a notícias e informações sobre COVID-19 pelo rádio										
Nenhuma vez	2.180	47,6	1.304	38,4	<0,001	1.656	51,1	1.828	38,6	<0,001
Poucas vezes e algumas vezes	1.838	40,1	1.516	44,7		1.190	36,7	2.164	45,7	
Frequentemente	563	12,3	575	16,9		396	12,2	742	15,7	
**Horas de exposição a notícias e informações sobre COVID-19 pelo rádio**										
0 h	2.295	50,1	1.341	39,5	<0,001	1.779	54,9	1.857	39,2	<0,001
1 h e mais	2.285	49,9	2.054	60,5		1.462	45,1	2.877	60,8	

*p = Teste U de Mann-Whitney; ^†^População do estudo; ^‡^N = Distribuição dos participantes em cada resposta; ^§^% = Porcentagem em relação ao total de respostas na coluna referente a cada variável

Na análise multivariada, as seguintes variáveis apresentaram p-valor < 0,05 na regressão independente dos blocos, para rastreio de ansiedade: bloco 1, país de origem; bloco 2, sexo, faixa etária, estado civil, raça/cor (não branco) , quantidade de moradores no domicílio, área de residência, escolaridade (do ensino médio para mais) e alteração da renda durante a pandemia por COVID-19 (diminuição da renda); bloco 3, frequência (algumas vezes) e horas (duas horas ou mais) de exposição a notícias e informações sobre COVID-19 em redes sociais, uso e horas (uma hora) de exposição a notícias e informações sobre COVID-19 pela televisão, e uso, frequência (frequentemente) e nenhuma hora de exposição a notícias e informações sobre COVID-19 pelo rádio.

Contudo, no modelo final (Tabela 4), com R² de Nagelkerke = 0,312 e Teste de Hosmer e Lemeshow p-valor <0,001, permaneceram significativas as seguintes variáveis: país de origem, sexo (masculino), estado civil, raça (não branco), alteração da renda durante a pandemia por COVID-19, frequência (frequentemente) e horas (duas horas ou mais) de exposição a notícias e informações sobre COVID-19 em redes sociais, uso da televisão e horas (uma hora) para acessar notícias e informações sobre COVID-19, e frequência (algumas vezes) de exposição a notícias e informações sobre COVID-19 pelo rádio.

**Tabela 4-:** Modelo final de rastreamento ou não para ansiedade geriátrica e valor de p* das características socioeconômicas e demográficas, e a exposição a notícias e informações sobre COVID-19 de pessoas idosas (n^†^= 7.976). Peru, Brasil, México, 2022

Variável	Exposição (B) ajustada no bloco (IC95%) ^‡^	p*	Exposição (B) ajustada no modelo final (IC95%) ^‡^	p*
**Bloco 1 - Variável de origem dos participantes**				
**País**				
Peru	Ref ^§^			
Brasil	0,13	<0,001	0,10	<0,001
	0,11-0,14		0,09-0,12	
México	0,05	<0,001	0,05	<0,001
	0,03-0,07		0,03-0,08	
**Bloco 2 - Variáveis socioeconômicas e demográficas**				
**Sexo biológico**				
Feminino	Ref ^§^			
Masculino	1,13	0,018	1,13	0,031
	1,02-1,25		1,01-1,26	
Prefiro não declarar	0,85	0,761	2,46	0,079
	0,31-2,38		0,90-6,72	
**Faixa etária (anos)**				
60-64	Ref ^§^			
65-69	1,14	0,043	1,07	0,300
	1,00-1,29		0,94-1,23	
70-74	1,18	0,031	0,99	0,900
	1,02-1,37		0,84-1,16	
75-79	1,26	0,006	1,05	0,577
	1,07-1,49		0,88-1,27	
80 ou acima	1,45	<0,001	1,04	0,690
	1,22-1,73		0,86-1,26	
**Estado civil**				
Solteiro(a)	Ref ^§^			
Casado(a)/morando junto	0,61	<0,001	0,68	<0,001
	0,52-0,73		0,57-0,82	
Separado(a)/desquitado(a)	0,63	<0,001	0,74	0,009
	0,51-0,78		0,59-0,93	
Viúvo(a)	0,70	<0,001	0,72	0,002
	0,58-0,84		0,59-0,89	
**Raça/cor**				
Branco	Ref ^§^			
Não branco	3,63	<0,001	1,39	<0,001
	3,26-4,05		1,21-1,59	
**Quantidade de moradores no domicílio**				
Mora sozinho	Ref ^§^			
Uma ou duas	1,26	0,017	1,06	0,563
	1,04-1,51		0,87-1,30	
Três ou mais	1,76	<0,001	1,02	0,886
	1,45-2,12		0,82-1,25	
**Residência própria**				
Não	Ref ^§^			
Sim	1,10	0,146	1,13	0,073
	0,97-1,24		0,99-1,30	
Área de residência				
Urbana	Ref ^§^			
Rural	1,34	<0,001	0,92	0,257
	1,17-1,54		2,79-1,06	
**Educação m** áxima				
Não estudou ou não concluiu o ensino básico	Ref ^§^			
Ensino básico ou fundamental	1,15	0,139	0,92	0,420
	0,95-1,39		0,75-1,13	
Ensino médio	1,60	<0,001	1,04	0,754
	1,32-1,94		0,83-1,28	
Ensino superior completo	2,21	<0,001	1,22	0,082
	1,82-2,68		0,97-1,53	
Outros	1,28	0,039	1,21	0,144
	1,01-1,61		0,94-1,58	
**Alteração da renda durante a pandemia por COVID-19a**				
Não	Ref ^§^			
Sim, a minha renda aumentou	1,06	0,671	0,71	0,021
	0,81-1,39		0,53-0,95	
Sim, a minha renda diminuiu	1,12	0,029	0,72	<0,001
	1,01-1,24		0,64-0,81	
**Bloco 3 - Variáveis de exposição a notícias e informações sobre COVID-19**				
**Utiliza redes sociais para acessar notícias e informações sobre COVID-19**				
Não	Ref ^§^			
Sim	1,05	0,448	0,98	0,819
	0,92-1,20		0,85-1,14	
**Frequência de exposição na última semana a notícias e informações sobre COVID-19 em redes sociais**				
Nenhuma vez	Ref ^§^			
Poucas vezes e algumas vezes	0,77	0,002	1,13	0,217
	0,65-0,91		0,93-1,36	
Frequentemente	0,94	0,528	1,59	<0,001
	0,76-1,15		1,25-2,03	
**Horas de exposição a notícias e informações sobre COVID-19 em redes sociais**				
0 h	Ref ^§^			
1 h	1,14	0,137	1,04	0,710
	0,96-1,35		0,85-1,26	
2 h a 5 h	0,77	0,004	0,73	0,003
	0,64-0,92		0,59-0,90	
6 h ou mais	0,62	<0,001	0,87	0,324
	0,49-0,78		0,67-1,14	
**Utiliza televisão para acessar notícias e informações sobre COVID-19**				
Não	Ref ^§^			
Sim	1,20	0,010	1,20	0,031
	1,04-1,39		1,02-1,41	
**Frequência de exposição na última semana a notícias e informações sobre COVID-19 pela televisão**				
Nenhuma vez	Ref ^§^			
Poucas vezes e algumas vezes	1,07	0,492	0,97	0,810
	0,88-1,30		0,78-1,21	
Frequentemente	0,84	0,112	0,92	0,502
	0,67-1,04		0,71-1,18	
**Horas de exposição a notícias e informações sobre COVID-19 pela televisão**				
0 h	Ref ^§^			
1 h	0,78	0,013	0,70	0,002
	0,64-0,95		0,56-0,88	
2 h	0,98	0,818	0,97	0,782
	0,79-1,21		0,76-1,23	
3 h ou mais	0,83	0,094	0,84	0,162
	0,67-1,03		0,66-1,07	
**Utiliza rádio para acessar notícias e informações sobre COVID-19**				
Não	Ref ^§^			
Sim	1,30	<0,001	1,03	0,724
	1,14-1,48		0,88-1,20	
**Frequência de exposição na última semana a notícias e informações sobre COVID-19 pelo rádio**				
Nenhuma vez	Ref ^§^			
Poucas vezes e algumas vezes	1,01	0,852	0,77	0,003
	0,87-1,18		0,65-0,91	
Frequentemente	1,26	0,018	1,06	0,615
	1,04-1,53		0,85-1,32	
**Horas de exposição a notícias e informações sobre COVID-19 pelo rádio**				
0 h	Ref ^§^			
1 h e mais	1,22	0,010	0,85	0,067
	1,05-1,42		0,71-1,01	

*p = p-valor; ^†^População do estudo; ^‡^(IC 95%) = Intervalo de confiança de 95%; ^§^Ref = Categoria de referência

Para o rastreio de depressão, na regressão independente dos blocos, as variáveis que apresentaram diferenças estatisticamente significativas foram: bloco 1, país de origem; bloco 2, sexo (masculino), faixa etária, estado civil (separado/desquitado), raça/cor (não branco), quantidade de moradores no domicílio, área de residência, escolaridade (outras) e alteração da renda durante a pandemia por COVID-19 (diminuição da renda); bloco 3, uso, frequência (algumas vezes e frequentemente) e horas (uma hora ou mais) de exposição a notícias e informações sobre COVID-19 em redes sociais, uso e horas (uma hora ou mais) de exposição a notícias e informações sobre COVID-19 pela televisão, e uso e horas (uma hora ou mais) de exposição a notícias e informações sobre COVID-19 pelo rádio.

No modelo final (Tabela 5), com R² de Nagelkerke = 0,169 e Teste de Hosmer e Lemeshow p-valor <0,001, as seguintes variáveis apresentaram p-valor < 0,05: país de origem, sexo (masculino), faixa etária (80 anos ou mais), raça (não branco), escolaridade (do ensino básico ou fundamental para mais), alteração da renda durante a pandemia por COVID-19 (diminuição da renda), uso e horas (uma hora ou mais) de exposição a notícias e informações sobre COVID-19 pela televisão, e horas (uma hora ou mais) de exposição a notícias e informações sobre COVID-19 pelo rádio.

**Tabela 5-:** Modelo final de rastreamento ou não para depressão geriátrica e valor de p* das características socioeconômicas e demográficas, e a exposição a notícias e informações sobre COVID-19 de pessoas idosas (n^†^= 7.976). Peru, Brasil, México, 2022

Variável	Exposição (B) ajustada no bloco (IC 95%) ^‡^	p*	Exposição (B) ajustada no modelo final (IC95%) ^‡^	p*
**Bloco 1 - Variável de origem dos participantes**				
**País**				
Peru	Ref ^§^			
Brasil	0,23	<0,001	0,23	<0,001
	0,21-0,26		0,19-0,26	
México	0,49	<0,001	0,45	<0,001
	0,39-0,63		0,34-0,60	
**Bloco 2 - Variáveis socioeconômicas e demográficas**				
**Sexo biológico**				
Feminino	Ref ^§^			
Masculino	0,88	0,014	0,83	0,001
	0,80-0,98		0,75-0,92	
Prefiro não declarar	1,29	0,610	2,07	0,138
	0,49-3,41		0,79-5,44	
**Faixa etária (anos)**				
60-64	Ref ^§^			
65-69	1,26	<0,001	1,17	0,012
	1,12-1,43		1,04-1,33	
70-74	1,26	0,002	1,09	0,241
	1,09-1,46		0,94-1,27	
75-79	1,29	0,003	1,15	0,110
	1,09-1,51		0,97-1,37	
80 ou acima	1,70	<0,001	1,43	<0,001
	1,43-2,03		1,18-1,72	
**Estado civil**				
Solteiro(a)	Ref ^§^			
Casado(a)/morando junto	0,98	0,775	1,08	0,405
	0,83-1,15		0,91-1,28	
Separado(a)/desquitado(a)	0,77	0,010	0,87	0,200
	0,63-0,94		0,71-1,07	
Viúvo(a)	1,02	0,864	1,05	0,647
	0,85-1,22		0,86-1,26	
**Raça/cor**				
Branco	Ref ^§^			
Não branco	1,62	<0,001	0,86	0,022
	1,47-1,80		0,76-0,98	
**Quantidade de moradores no domicílio**				
Mora sozinho	Ref ^§^			
Uma ou duas	1,19	0,043	1,05	0,583
	1,01-1,41		0,88-1,25	
Três ou mais	1,46	<0,001	0,96	0,655
	1,22-1,74		0,80-1,15	
**Residência própria**				
Não	Ref ^§^			
Sim	0,93	0,259	0,93	0,246
	0,82-1,05		0,81-1,05	
Área de residência				
Urbana	Ref ^§^			
Rural	1,46	<0,001	1,09	0,283
	1,26-1,69		0,93-1,27	
**Educação m** áxima				
Não estudou ou não concluiu o ensino básico	Ref ^§^			
Ensino básico ou fundamental	0,91	0,337	0,76	0,007
	0,75-1,10		0,62-0,93	
Ensino médio	1,00	0,990	0,71	0,001
	0,82-1,21		0,58-0,88	
Ensino superior completo	1,12	0,246	0,71	0,002
	0,92-1,36		0,57-0,88	
Outros	0,70	0,001	0,66	<0,001
	0,56-0,87		0,52-0,83	
**Alteração da renda durante a pandemia por COVID-19**				
Não	Ref ^§^			
Sim, a minha renda aumentou	1,26	0,084	0,97	0,815
	0,97-1,63		0,74-1,27	
Sim, a minha renda diminuiu	1,66	<0,001	1,19	0,001
	150-1,83		1,07-1,33	
**Bloco 3 - Variáveis de exposição a notícias e informações sobre COVID-19**				
**Utiliza redes sociais para acessar notícias e informações sobre COVID-19**				
Não	Ref ^§^			
Sim	1,15	0,037	1,13	0,078
	1,01-1,31		0,99-1,30	
**Frequência de exposição na última semana a notícias e informações sobre COVID-19 em redes sociais**				
Nenhuma vez	Ref ^§^			
Poucas vezes e algumas vezes	0,81	0,015	1,12	0,216
	0,69-0,96		0,94-1,34	
Frequentemente	0,68	<0,001	1,00	0,968
	0,55-0,84		0,80-1,26	
**Horas de exposição a notícias e informações sobre COVID-19 em redes sociais**				
0 h	Ref ^§^			
1 h	0,82	0,026	0,86	0,114
	0,69-0,98		0,71-1,04	
2 h a 5 h	0,82	0,031	0,92	0,398
	0,68-0,98		0,76-1,12	
6 h ou mais	0,76	0,017	1,08	0,529
	0,60-0,95		0,85-1,38	
**Utiliza televisão para acessar notícias e informações sobre COVID-19**				
Não	Ref ^§^			
Sim	0,79	0,001	0,86	0,047
	0,68-0,91		0,73-1,00	
**Frequência de exposição na última semana a notícias e informações sobre COVID-19 pela televisão**				
Nenhuma vez	Ref ^§^			
Poucas vezes e algumas vezes	1,01	0,940	0,91	0,362
	0,83-1,23		0,74-1,12	
Frequentemente	0,88	0,244	0,94	0,590
	0,70-1,09		0,74-1,19	
**Horas de exposição a notícias e informações sobre COVID-19 pela televisão**				
0 h	Ref ^§^			
1 h	1,44	<0,001	1,36	0,005
	1,18-1,76		1,10-1,68	
2 h	1,41	0,002	1,36	0,008
	1,13-1,75		1,09-1,72	
3 h ou mais	1,59	<0,001	1,66	<0,001
	1,28-1,97		1,32-2,09	
**Utiliza rádio para acessar notícias e informações sobre COVID-19**				
Não	Ref ^§^			
Sim	1,23	0,002	1,08	0,305
	1,08-1,41		0,93-1,25	
**Frequência de exposição na última semana a notícias e informações sobre COVID-19 pelo rádio**				
Nenhuma vez	Ref ^§^			
Poucas vezes e algumas vezes	1,05	0,541	0,90	0,217
	0,90-1,22		0,77-1,06	
Frequentemente	1,06	0,539	0,98	0,868
	0,87-1,29		0,80-1,21	
**Horas de exposição a notícias e informações sobre COVID-19 pelo rádio**				
0 h	Ref ^§^			
1 h e mais	1,49	<0,001	1,21	0,020
	1,28-1,74		1,03-1,43	

*p = p-valor; ^†^População do estudo; ^‡^(IC 95%) = Intervalo de confiança de 95%; ^§^Ref = Categoria de referência

## Discussão

Este estudo verificou a associação entre exposição a notícias e informações sobre COVID-19 e o impacto na saúde mental de pessoas idosas do Peru, Brasil e México. A prevalência para o rastreio de ansiedade geriátrica dentre os participantes foi de 42,6%. Distingue-se que, somente na amostra de pessoas idosas do Peru, a prevalência foi majoritária para o desfecho, com 63,5%. Para sintomas depressivos, 59,4% da amostra indicou o rastreio, sendo que nas amostras dos participantes peruanos a frequência foi de 74,1% e na amostra mexicana, 58,6%.

Os achados estão acima da frequência observada em revisão sistemática^(13)^ focalizada na América Latina, que estima 35% (IC 95%: 31–38%) da prevalência para ansiedade e 35% (IC 95%: 31–39%) para sintomas depressivos. Quando observado outras regiões do mundo^(11)^, para sintomas ansiosos, a prevalência varia de 7,4% a 47,82% e para depressão, de 14,14% a 48,3%. Contudo, limita-se que as revisões não possuem a amostra exclusivamente de pessoas idosas e os instrumentos utilizados para rastreio dos sintomas distinguem-se dos aplicados no estudo. No mais, o início do período da coleta de dados coincidiu com o primeiro maior pico de mortes por COVID-19 registrado nas Américas, em destaque para o dia 18 de janeiro de 2021, podendo também ter contribuído para os achados distintos da literatura^(1)^.

Para o uso das mídias no acesso a notícias e informações sobre COVID-19, os participantes indicaram 77,6% o consumo pela televisão, 46,6% o uso das redes sociais e 42,7% a referência ao rádio para essa finalidade. Somente para a amostra de pessoas idosas brasileiras, houve prevalência do uso das redes sociais, com 58,8%. Apenas a amostra de peruanos refere o consumo pelo rádio em 57,4%. Distinguem-se os participantes mexicanos pelo não uso da televisão, em frequência 54,8%, além de não apresentarem frequência majoritariamente positiva para nenhuma das três mídias.

Percebe-se que em nenhum dos itens houve concordância dos três países. Em análise do jornalismo mundial no começo de 2023, o acesso à internet no Peru, Brasil e México é apontado, respectivamente, em 87%, 83% e 67%, e o acesso *online* (incluindo redes sociais) é a principal fonte de acesso a notícias, ainda que seja percebida uma diminuição geral nesse consumo. Essa redução também é identificada nas mídias tradicionais, como a televisão e os jornais impressos^(23)^.

No Brasil, o consumo de notícias *online* (incluindo redes sociais) diminuiu de 90% para 79% nos últimos 10 anos, com destaque para o uso do *WhatsApp*. A televisão é indicada por 51% dos participantes. Em semelhança, o Peru e o México apresentam o consumo de notícias *online* (incluindo redes sociais) em 80%, principalmente pelo *Facebook*. Diferenciam-se em relação à televisão, sendo o consumo em 51% no Peru e 42% no México^(23)^.

A distinção dos achados entre os países inspira a acentuar o contexto desses territórios e a compreender o comportamento informacional da população amostral exclusivamente de pessoas idosas. Observa-se que os participantes se envolveram com notícias e informações sobre COVID-19, inclusive pelas mídias digitais (redes sociais). Todavia, a qualidade das informações, o nível de confiança e a literacia das pessoas idosas não foram mensurados.

Em estudo pré-pandemia sobre o acesso a meios digitais de pessoas idosas em alguns dos países latino-americanos, dentre eles, Peru e México, as pessoas idosas compuseram menos de 10% da população usuária, com destaque ao uso para informação, seguido pela comunicação^(24)^. Já na pandemia^(8)^, apontam que no Peru, a incapacidade de reconhecer notícias falsas alcançou 79% da população. Acima do México, com 66%, e do Brasil, em 62%.

Combina-se à baixa literacia digital de pessoas idosas^(25)^ e a níveis declinantes de confiança da população nas notícias, oriundos, principalmente, da situação política e do comportamento de seus presidentes, a saber, o Brasil com 43% de confiança, seguido do México com 36% e o Peru, 33%^(23)^.

Na nossa amostra, a associação bivariada do uso das mídias para o desfecho rastreamento de ansiedade apresentou diferenças estatisticamente significativas para o uso das redes sociais (X² = 43,6 e p-valor <0,001) e o rádio (X² = 135,0 e p-valor <0,001). Contudo, somente para o rádio houve frequência de 50% para a ocorrência ou não do desfecho. Relativo ao rastreio de depressão, para as redes sociais (X² = 29,0 e p-valor < 0,001), televisão (X² = 3,83 e p-valor < 0,050) e rádio (X² = 138,0 e p-valor < 0,001), a frequência para o desfecho foi maior independente do uso.

O impacto das mídias sociais digitais na percepção de solidão e no isolamento social de pessoas idosas foi analisado em revisão integrativa, que sinalizou o uso dessas mídias com potencial para reduzir sentimentos de solidão e isolamento social, promovendo maior interação e senso de pertencimento entre os idosos^(26)^.

Esses achados convergiram parcialmente com os resultados do presente estudo, uma vez que também se identificou associação significativa entre exposição às mídias sociais e alterações na saúde mental dos participantes durante a pandemia. No entanto, diferentemente dos resultados encontrados na revisão integrativa, nosso estudo indicou que a exposição frequente às mídias sociais esteve associada a maior prevalência de sintomas ansiosos e depressivos, sugerindo que o contexto pandêmico pode ter influenciado negativamente essa relação^(26)^.

Dessa maneira, os achados sinalizam para pessoas idosas conectadas às informações sobre COVID-19 e passíveis de rastreio para transtornos mentais, com destaque para a depressão, mas que, no limite do método e das análises, não é possível inferir a direção das reações considerando o contexto conhecido dos participantes.

No modelo final para rastreio de ansiedade geriátrica, com explicação da variação em 31,18% pelo R² de Nagelkerke e p-valor de Hosmer e Lemeshow <0,001, controlado por fatores socioeconômicos, estima-se que pessoas idosas participantes do Brasil tiveram 0,10 (IC 95% 0,09 a 0,12 e p-valor < 0,001) vezes a chance de rastreio para ansiedade. Em relação às pessoas idosas do México, 0,05 (IC 95% 0,03 a 0,08 e p-valor < 0,001) vezes a chance, em relação à categoria de referência — as pessoas idosas do Peru.

Sobre a exposição a notícias e informações sobre COVID-19, as três mídias apresentaram diferentes significâncias. O uso das redes sociais para essa finalidade apontou 1,59 (IC 95% 1,25 a 2,03 e p-valor <0,001) vezes a chance para o rastreio de ansiedade geriátrica em relação a quem não utilizou dessa maneira. Em semelhante, de 2 a 5 horas de consumo pelas redes sociais, apresentou 0,73 (IC 95% 0,59 a 0,90 e p-valor de 0,003) vezes a chance para o rastreio em relação a quem não se expôs hora nenhuma.

Em relação à televisão, o uso para informação sobre COVID-19 significou 1,20 (IC 95% 1,02 a 1,41 e p-valor de 0,031) vezes a chance para o desfecho em relação a quem não utiliza. Sendo uma hora, 0,70 (IC 95% 0,56 a 0,88 e p-valor de 0,002) vezes a chance para rastreio em relação a quem não tem nenhuma hora de exposição com essa intenção. O rádio, somente a frequência, uma ou duas vezes na semana, indica 0,77 (IC 95% 0,65 a 0,91 e p-valor de 0,003) vezes a chance para o desfecho, sobre quem não se expôs para informação sobre COVID-19.

O segundo modelo, com desfecho para depressão, com explicação da variação em 16,89% pelo R² de Nagelkerke e p-valor de Hosmer e Lemeshow <0,001, controlado por fatores socioeconômicos, os brasileiros apresentaram 0,23 (IC 95% 0,19 a 0,26 e p-valor < 0,001) vezes a chance de rastreio e os mexicanos 0,45 (IC 95% 0,34 a 0,60 e p-valor < 0,001) vezes a chance de rastreio, em relação às pessoas idosas peruanas.

Somente para televisão e rádio verificaram-se significâncias para o desfecho. Sendo, três horas ou mais de exposição a notícias e informações sobre COVID-19 pela televisão 1,66 (IC 95% 1,32 a 2,09 e p-valor < 0,001) vezes sobre quem não se expôs nenhuma hora para esse fim. O tempo de exposição também foi relevante em relação ao rádio, uma hora ou mais apontou 1,21 (IC 95% 1,03 a 1,43 e p-valor 0,020) vezes em relação a quem não consumiu por essa mídia.

Estes resultados reafirmam a associação do consumo midiático (digital ou não) de informação sobre a COVID-19 com a possibilidade de rastreio para os transtornos mentais analisados de pessoas idosas participantes. Em nenhuma das variáveis associadas, a ausência ou o baixo consumo de uma das mídias indicou maior possibilidade dos desfechos.

Em revisão sistemática^(27)^, o uso das mídias sociais insere-se nas causas da infodemia e dentre os impactos, apontam-se as questões psicológicas. O ambiente oportuno de circulação das desinformações (nas variadas possibilidades) foram as redes sociais e, inevitavelmente, extravasou. Em um “círculo vicioso da infodemia”^(27)^, o impacto na saúde mental amplifica a máquina infodêmica que retorna no agravamento dos transtornos.

No movimento de gestão da infodemia, discute-se a desigualdade de acesso a informações de qualidade, atualizadas e coerentes com as necessidades dos indivíduos e das comunidades. No potencial danoso à saúde, os estados emocionais, decorrentes da sobrecarga de informação, podem não iniciar com as informações, mas envolver estados previamente existentes, com capacidade de influenciar o comportamento dos indivíduos^(6)^.

Delimitado pelo objetivo do artigo, não é possível permear todas as nuances da complexidade da informação com a saúde, mas coaduna-se com a discussão^(28)^ que a sobrecarga de informação não é um processo agudo, mas estrutural, que integra a determinação social da saúde. A desigualdade informativa, a infodemia, o etarismo, os monopólios midiáticos e o modelo manicomial de saúde operam no mesmo universo de comercialização da vida. O rompimento dessas estruturas prevê não somente o antagonismo, mas a construção de projeto de sociedade que inclua também a garantia de direito à informação de qualidade, gratuita, segura e acessível.

Este estudo apresenta, como limitação, o esforço de utilizar os mesmos instrumentos de rastreio para ansiedade e depressão geriátrica nos três países, o que pode ter influenciado nas prevalências. Além disso, como próprio de estudos observacionais transversais, não é possível aferir se a relação cronológica da coleta de dados e da pandemia por COVID-19 modificaria os achados. No mais, sabe-se da possibilidade de vieses de seleção e informação, na coleta de dados por *web-based survey*, em amostra não probabilística, restringindo a generalização dos achados.

Esta pesquisa contribui significativamente para o avanço científico ao demonstrar diferenças transnacionais na prevalência de ansiedade e depressão geriátrica associadas ao consumo midiático durante a pandemia entre pessoas idosas no Peru, Brasil e México. Os achados são relevantes para a prática da enfermagem gerontológica nesses países, por orientarem estratégias específicas de promoção da saúde mental e literacia em saúde adaptadas às realidades locais.

## Conclusão

Verificou-se associação entre a exposição frequente às mídias com a maior prevalência de ansiedade e depressão geriátrica nas pessoas idosas estudadas. A diferença significativa da prevalência para o desfecho, em comparação aos três países, foi pequena. Esses resultados reforçam a necessidade de estratégias específicas na gestão da infodemia para proteção da saúde mental das pessoas idosas na América Latina.

Visto as limitações existentes no estudo, as pessoas idosas dos países latino-americanos analisados estão em concordância com a literatura que indica que essa população está presente e adotando as mídias digitais (nesse estudo, com foco nas redes sociais). Além disso, a saúde mental das pessoas idosas, que se estende às relações com a informação, corrobora os achados da demanda e necessidade de cuidado e políticas públicas direcionadas. Diante disso, reforça-se que não se tem a pretensão de delimitar e reforçar estereótipos biomédicos da vivência e relação dos indivíduos com transtornos mentais, uma possível sintomatologia isoladamente não determina comportamentos e escolhas dos indivíduos.

A pesquisa oportuniza agregar ao conhecimento produzido e centrado na América-Latina, com articulação atual, vinculado à pandemia por COVID-19 e implicações futuras, com articulação ao envelhecimento populacional e à gestão da infodemia enquanto também política pública de saúde. Espera-se contribuir para a realização de pesquisas que se agregam ao direito à informação e ao envelhecimento saudável. É preciso investir em prevenção e promoção da saúde por meio da educação ― literacia em saúde ― e da participação ― com escuta às demandas e as necessidades em saúde ― para a construção e/ou fortalecimento de políticas públicas intersetoriais e conscientes da potência dos povos latino-americanos.

# Translation: Prevalencia transnacional de trastornos mentales en adultos mayores expuestos a información sobre COVID-19*

**DOI:** 10.1590/1518-8345.7580.4631

**Authors:** Daniele Knopp Ribeiro, Fábio da Costa Carbogim, Patricia Rodrigues Braz, Sofia Sabina Lavado-Huarcaya, Aracely Díaz-Oviedo, Alexandre Favero Bulgarelli, Rosimere Ferreira Santana, Ione Carvalho Pinto, Fabiana Costa Machado Zacharias, Ricardo Bezerra Cavalcante

**Affiliations:** 1 Universidade Federal de Juiz de Fora, Juiz de Fora, MG, Brasil.; 2 Becaria de la Coordenação de Aperfeiçoamento de Pessoal de Nível Superior (CAPES), Brasil.; 3 Universidad Señor de Sipán, Escuela de Postgrado, Pimentel, Lambayeque, Peru.; 4 Universidad Autónoma de San Luís Potosí, Facultad de Enfermería y Nutrición, San Luis Potosí, SLP, Mexico.; 5 Universidade Federal do Rio Grande do Sul, Porto Alegre, RS, Brasil.; 6 Universidade Federal Fluminense, Faculdade Enfermagem, Niterói, RJ, Brasil.; 7 Universidade de São Paulo, Escola de Enfermagem de Ribeirão Preto, Centro Colaborador de la OPS/OMS para el Desarrollo de la Investigación en Enfermería, Ribeirão Preto, SP, Brasil.

**Keywords:** Infodemia, Salud Mental, Anciano, América Latina, Covid-19, Estudios Transversales

## Abstract

verificar la asociación entre la exposición a noticias e información sobre COVID-19, a través de redes sociales, televisión y radio, y la detección de ansiedad y depresión geriátrica comparando Perú, Brasil y México.

diseño transversal, mediante *web-based survey*, en una muestra no probabilística, con escalas validadas para la detección de ansiedad y depresión geriátrica, y análisis de datos mediante regresión logística binaria jerárquica.

de los 7.976 participantes, la mayoría era del sexo femenino (n=4.937, 61,9%), de color/raza no blanca (n=4.724, 59,2%) y pertenecía a la franja etaria de 60 a 64 años (n=2.584, 32,4%). El acceso a noticias e información sobre COVID-19 se produjo mediante la televisión (n=6.187, 77,6%), pocas o algunas veces por semana (n=4.322, 54,2%) durante tres horas o más (n=2.596, 32,5%). En los modelos finales, para ansiedad o depresión, las diferencias significativas (valor p<0,001) de los aspectos uso, frecuencia y horas de exposición variaron dependiendo del medio. La prevalencia de los resultados, al comparar los tres países, fue pequeña.

la exposición frecuente a los medios de comunicación se asoció con una mayor prevalencia de ansiedad y depresión geriátrica, aunque la diferencia entre los países estudiados fue pequeña.

## Destacados

**(1)** Las variables asociadas a los resultados indican el consumo de noticias e información. **(2)** La frecuencia y el tiempo de exposición a los medios son importantes para la detección. **(3)** La prevalencia del resultado fue pequeña al comparar los tres países. **(4)** La detección de ansiedad fue del 42,6% y la de depresión del 59,4%. **(5)** El consumo de los medios fue 77,6% televisión, 46,6% redes sociales y 42,7% radio.

## Introducción

El 30 de enero de 2020 y posteriormente el 11 de marzo del mismo año, se reconoció el riesgo para la salud pública y la capacidad de contagio del coronavirus SARS-CoV-2, responsable de la enfermedad COVID-19. Aproximadamente cuatro años después, se notificaron más de 772 millones de casos y aproximadamente 7 millones de muertes en todo el mundo, los continentes europeo y americano se destacaron en la prevalencia de casos y muertes notificadas, respectivamente^(1)^.

La salud de la población de América Latina y el Caribe fue la más afectada, a nivel mundial, por la pandemia de COVID-19. A pesar de las diferentes medidas que se tomaron en los sistemas de salud y la diversidad social y política, los grupos históricamente vulnerables, como las mujeres, las personas no blancas, las personas en situación de pobreza y/o calle, los estudiantes y los adultos mayores, vivieron la pandemia de manera diferente y sufrieron el agravamiento de las desigualdades históricas en la región^(2-3)^.

La complejidad de la experiencia de la pandemia se incrementó por el exceso y la velocidad de la información producida y difundida en los medios de comunicación masivos tradicionales, y aumentó aún más debido a las redes sociales. En este contexto, se tarda en reconocer el origen, la intención y la calidad de la información, y a este fenómeno se lo denomina infodemia^(4)^. Esto puede provocar cambios en la percepción del riesgo, sentimientos y sensaciones de confusión y desorientación, reticencia, parálisis, negación, desconfianza, entre otros, que se relacionan con las experiencias previas de los individuos y con personas de confianza, el sistema de salud y el gobierno de la región^(5-6)^.

En menos de un año de haberse reconocido la pandemia, se informó la rudimentaria estructura y coordinación de los gobiernos latinoamericanos para manejar la infodemia^(7)^, en base al análisis de los sitios *web* de los ministerios de salud de 10 países. Paralelamente^(8)^, en un estudio ecológico descriptivo, se destaca que, en seis países latinoamericanos estudiados, los lugares con mayor dificultad para reconocer noticias falsas y el papel central que juegan las redes sociales como medio de información coincidieron con las mayores tasas de mortalidad por COVID-19 en ese período.

Además, en los medios de comunicación de Brasil y Chile en 2020/2021 se encontraron discursos del tipo “ya vivieron su vida”, que culpaban a la gravedad de la pandemia y a la homogeneidad de las muertes, cuando se referían a las personas mayores. Aunque también se reconoce el posible empeoramiento de la salud mental después de la pandemia, producto del ridículo, abandono, dependencia y control al que fue sometida esa población^(9)^.

El agravamiento de la vulnerabilidad de las personas mayores va más allá de la histórica condición socioeconómica-demográfica de inequidades, y se suma al conocido aumento del envejecimiento poblacional que durante la pandemia de COVID-19 se refleja en la tasa de mortalidad proporcional al aumento de la edad^(1-2)^. La necesidad de investigar, manejar y fortalecer las políticas públicas forma parte del movimiento por la Década del Envejecimiento Saludable 2021-2030, que en las Américas es coordinado por la Organización Panamericana de la Salud (OPS)^(10)^.

La realización del presente estudio se justifica por la escasez de estudios que incluyan exclusivamente poblaciones de adultos mayores y/o las implicancias para la salud mental derivadas de la infodemia en países de América Latina, como Perú, Brasil y México. Si bien se considera que síntomas complejos como ansiedad, depresión e insomnio, durante la pandemia, se informan en revisiones sistemáticas con población adulta en diferentes regiones del mundo, cuando se asocian a los medios de comunicación, el foco está principalmente en los medios de comunicación sociales^(11-13)^.

Por lo tanto, se busca verificar la asociación entre la exposición a noticias e información sobre COVID-19, a través de redes sociales, televisión y radio, y la detección de ansiedad y depresión geriátrica comparando adultos mayores de Perú, Brasil y México.

## Método

### Diseño del estudio

Estudio transversal, realizado con adultos mayores de 60 años, entre julio de 2020 y junio de 2022, en Perú, Brasil y México. Derivado de la parte 1 de la investigación “Infodemia COVID-19 y sus repercusiones en la salud mental de las personas mayores durante y después de la pandemia: estudio multicéntrico en Brasil, Perú, Colombia, México y Portugal”, que tiene como objetivo “analizar la relación entre la infodemia COVID-19 y las repercusiones en la salud mental de las personas mayores”.

### Aspectos éticos

El estudio fue aprobado por el Comité de Ética en Investigación con Humanos de Brasil (CAAE: 31932620.1.1001.5147, bajo dictamen n° 4.134.050), Perú y México (Universidad Autónoma de San Luis Potosí, bajo registro CONBIOÉTICA-24-CEI-002-20230925).

### Criterios de selección

Se incluyeron personas mayores de 60 años, con capacidad cognitiva conservada, acceso a *e-mail* y/o redes sociales y/o teléfono. Fueron excluidas las personas mayores que, según el autoinforme de la persona contactada, no estaban en condiciones de responder las preguntas de forma independiente.

### Escenarios

Los participantes residen en una de las siguientes ciudades: Brasil - Brasilia, Divinópolis, Juiz de Fora, Porto Alegre, Río de Janeiro, Ribeirão Preto, São Paulo y Viçosa; México - Cuidado Valles, Matehuala, Rioverde, Salinas de Hidalgo, San Luis Potosí, Soledas de Graciano Sánchez y Tamazunchale; Perú - Arequipa, Cerro de Pasco, Chiclayo, Huánuco, Iquitos, Lima, Puno- Juliaca, Tacna, Tarapoto, Tumbes y Trujillo.

### Recolección de datos

La recolección de datos se realizó mediante *web-based survey*, en una muestra no probabilística, difundida por *e-mail*, redes sociales (*WhatsApp*, *Facebook* e *Instagram*) y difundida por sociedades científicas de geriatría y gerontología, instituciones de salud, asociaciones de jubilados y centros de investigación. Se realizaron 15 entrevistas piloto en cada país para adaptar el idioma a las preguntas.

El *link* para acceder al cuestionario lleva al Formulario de Consentimiento Libre e Informado (FCLI), sólo los participantes que dieron su consentimiento tuvieron acceso a las preguntas, todas ellas figuraban como obligatorias. Todos recibieron una copia del FCLI firmada por el investigador a través de *e-mail* o redes sociales. También se contactó telefónicamente a las personas mayores, para invitarlos a participar de la investigación, en el momento de la llamada o al reprogramar, se les leyó el FCLI y se les indicó que dieran su consentimiento para grabar la llamada telefónica. Posteriormente, también se les envió el FCLI firmado por el investigador a través de *e-mail* o redes sociales.

### Instrumentos

El cuestionario estuvo compuesto por los siguientes segmentos: identificación, perfil sociodemográfico, exposición a noticias e información relacionada con la COVID-19^(14-15)^, impacto de los signos y síntomas en los trastornos psicopatológicos al estar en contacto con información sobre COVID-19 (investigación en desarrollo), Escala de Estrés Percibido, Inventario de Ansiedad Geriátrica (GAI)^(16)^ y Escala de Depresión Geriátrica (GDS-15)^(17)^. El presente estudio analiza el perfil sociodemográfico de los segmentos, la exposición a noticias e información relacionada con la COVID-19, GAI y GDS-15)^(16-17)^.

Para los datos recolectados en Brasil, el GAI^(18)^, validado con personas mayores del país, consta de 20 ítems de autorespuesta con preguntas binarias (de acuerdo/en desacuerdo) y un punto de corte <13 (no caso) y ≥13 (caso). La GDS-15^(19)^, también validada, contiene 15 preguntas, de las cuales 10 obtienen puntos si se responden positivamente y el resto si se responden negativamente, con un punto de corte <6 para no caso y ≥6 para caso.

Los datos recolectados en México siguen GAI^(20)^, versión validada en español con personas mayores de Madrid, debido a la falta de un estudio de validación en el país. El inventario consta de 20 ítems dicotómicos (de acuerdo/en desacuerdo), con un punto de corte de ≥11 para caso y <11 para no caso. GDS-15, validada con adultos mayores mexicanos^(21)^, consta de 15 preguntas con opciones con respuesta sí/no, de las cuales 10 obtienen puntos si se responden positivamente y el resto si se responden negativamente. Los autores no indican un punto de corte, se compararon puntajes altos (10 o más síntomas) y puntajes bajos (menos de cinco síntomas) para depresión. Se consideró ≥5 como caso y <5 como no caso.

En Perú se utilizó GAI^(20)^, versión validada en español con adultos mayores de Madrid, debido a la falta de un estudio de validación en el país. El inventario consta de 20 ítems dicotómicos (de acuerdo/en desacuerdo), con un punto de corte de ≥11 para caso y <11 para no caso. En la evaluación de los síntomas de depresión se utilizó el referencial validado para adultos mayores colombianos, compuesto por 15 ítems dicotómicos con respuesta sí/no, de las cuales 10 obtienen puntos si se responden positivamente y el resto si se responden negativamente, el punto de corte propuesto fue ≥5 para sospecha de depresión^(22)^.

Las variables independientes relacionadas con los resultados se organizaron en los siguientes bloques jerárquicos del modelo de análisis:

Bloque 1 - Variable de origen de los participantes: Perú, Brasil y México.

Bloque 2 - Variables socioeconómicas y demográficas: sexo; franja etaria; estado civil; raza/color de piel; vive con; situación de residencia; zona de residencia; máximo nivel educativo; cambio en los ingresos debido a la pandemia de COVID-19.

Bloque 3 - Variables de exposición a noticias e información sobre COVID-19: tipo de medio; frecuencia de exposición en la última semana; horas de exposición.

### Procesamiento y análisis de datos

En el análisis de los datos recolectados, los participantes recibieron un código numérico para mantener la confidencialidad, la tabulación se realizó en Hojas de cálculo de *Google* y enviada al *software* IBM *Statistical Package for the Social Sciences* (SPSS), versión 23.0 para *Windows*. Todas las variables fueron sometidas a análisis descriptivo, se calculó la distribución de frecuencias absolutas (n) y relativas (%). Para analizar las asociaciones se utilizó la prueba de chi-cuadrado (X^2^) de independencia de Pearson en el análisis bivariado de cada bloque para las variables independientes categóricas nominales, y la prueba U de Mann-Whitney para las variables independientes ordinales.

Seguido del análisis de regresión logística binaria jerárquica, en el que, inicialmente, todas las variables de los respectivos bloques conformaron análisis por separado. Para realizar la comparación, según el bloque fuente, las variables se introdujeron en el modelo multivariado final. Para todas las pruebas se consideró un nivel de significación del 5% y un intervalo de confianza del 95%.

## Resultados

De los 7.976 participantes, 4.377 (54,9%) procedían de Perú, 3.307 (41,5%) de Brasil y 292 (3,7%) de México. De esta muestra, 4.937 (61,9%) declararon ser mujeres y 4.724 (59,2%) informaron ser de color de piel/raza no blanca, que incluye negra, mulata, asiática e indígena. La franja etaria más frecuente fue la de 60 a 64 años, con 2.584 (32,4%) personas mayores. Con respecto al estado civil, 4.642 (58,2%) personas mayores declararon estar casadas o vivir en pareja.

En cuanto a la vivienda, 6.514 (81,7%) informaron tener vivienda propia, 6.830 (85,6%) viven en zona urbana y 3.744 (46,9%) viven con tres o más personas. Con respecto al nivel educativo más alto señalado, la educación superior fue la más frecuente, con 2.120 (26,6%) personas mayores. En relación a la influencia de la pandemia de COVID-19 en los ingresos, 4.112 (51,6%) participantes consideraron que no hubo cambios en los ingresos.

Respecto a la información sobre COVID-19 en las redes sociales, 4.259 (53,4%) participantes informaron no utilizar las redes sociales y 3.261 (40,9%) no informaron ninguna exposición en la semana anterior a la encuesta.

Para 6.187 (77,6%) participantes, la televisión (TV) fue el medio utilizado para acceder a noticias e información sobre COVID-19, sin embargo, 4.322 (54,2%) informaron haber visto televisión pocas veces o algunas veces en la semana anterior a la encuesta. Para 2.596 (32,5%) personas mayores, el uso de la televisión fue de tres horas o más por semana.

Respecto al uso de la radio, 4.568 (57,3%) manifestaron no haber accedido en ningún momento a noticias o información sobre COVID-19 a través de este medio en la semana previa a la encuesta. Los datos tabulados por país se pueden ver en la Tabla 1 a continuación:

**Tabla 1-:** Frecuencias según características socioeconómicas y demográficas, y exposición a noticias e información sobre COVID-19 de personas mayores (n* = 7.976). Perú, Brasil, México, 2022

**Variables**	**Países**							
	**Perú** **Frecuencia** **(n=4.377)** ^†^		**Brasil** **Frecuencia** **(n=3.307)** ^‡^		**México** **Frecuencia** **(n=292)** ^§^		**Frecuencia total** (n=7.976)*	
	**N** ^||^	**%** ^¶^	**N** ^||^	**%** ^¶^	**N** ^||^	**%** ^¶^	**N** ^||^	**%** ^¶^
**Bloque 2 - Variables socioeconómicas y demográficas**								
**Sexo biológico**								
Femenino	2.452	56,0	2.250	68,0	239	81,8	4.941	61,9
Masculino	1.925	44,0	1.039	31,4	53	18,2	3.017	37,8
Prefiero no declarar	0	0,0	18	0,5	0	0,0	18	0,2
**Franja etaria (años)**								
60-64	1.241	28,4	1.285	38,9	61	20,9	2.587	32,4
65-69	1.284	29,3	921	27,9	86	29,5	2.291	28,7
70-74	705	16,1	503	15,2	60	20,5	1.268	15,9
75-79	547	12,5	334	10,1	43	14,7	924	11,6
80 o más	600	13,7	264	8,0	42	14,4	906	11,4
**Estado civil**								
Soltero(a)	423	9,7	365	11,0	36	12,3	824	10,3
Casado(a)/en pareja	2.670	61,0	1.835	55,5	137	46,9	4.642	58,2
Divorciado(a)/separado(a)	322	7,4	509	15,4	23	7,9	854	10,7
Viudo(a)	962	22,0	598	18,1	96	32,9	1.656	20,8
**Raza/Color de piel**								
Blanco	607	13,9	2.364	71,5	281	96,2	3.252	40,8
No blanco	3.770	86,1	943	28,5	11	3,8	4.724	59,2
**Número de residentes en el hogar**								
Vive solo	189	4,3	587	17,8	33	11,3	809	10,1
Uno o dos	1.412	32,3	1.886	57,0	125	42,8	3.423	42,9
Tres o más	2.776	63,4	834	25,2	134	45,9	3.744	46,9
**Vivienda propia**								
No	872	19,9	551	16,7	39	13,4	1462	18,3
Sí	3.505	80,1	2.756	83,3	253	86,6	6.514	81,7
**Zona de residencia**								
Urbana	3.380	77,2	3.160	95,6	290	99,3	6.830	85,6
Rural	997	22,8	147	4,4	2	0,7	1.146	14,4
**Máximo nivel educativo**								
No estudió o no completó la educación básica	355	8,1	295	8,9	25	8,6	675	8,5
Educación básica o primaria	1.133	25,9	713	21,6	116	39,7	1.962	24,6
Educación secundaria	1.305	29,8	718	21,7	56	19,2	2.079	26,1
Educación superior completa	1.446	33,0	645	19,5	29	9,9	2.120	26,6
Otros	138	3,2	936	28,3	66	22,6	1.140	14,3
**Cambios en los ingresos durante la pandemia de COVID-19**								
No	1.536	35,1	2.437	73,8	139	47,6	4.112	51,6
Sí, mis ingresos aumentaron	172	3,9	80	2,4	15	5,1	267	3,3
Sí, mis ingresos disminuyeron	2.669	61,0	787	23,8	138	47,3	3.594	45,1
**Bloque 3 - Variables de exposición a noticias e información sobre COVID-19**								
Usa las redes sociales para acceder a noticias e información sobre COVID-19* *								
No	2.719	62,1	1.361	41,2	179	61,3	4.259	53,4
Sí	1.658	37,9	1.943	58,8	113	38,7	3.714	46,6
**Frecuencia de exposición en la última semana a noticias e información sobre COVID-19 en redes sociales**								
Ni una sola vez	2.280	52,1	822	24,9	159	54,5	3.261	40,9
Pocas veces y algunas veces	1.571	35,9	1.464	44,3	102	34,9	3.137	39,3
A menudo	526	12,0	1.021	30,9	31	10,6	1.578	19,8
Horas de exposición a noticias e información sobre COVID-19 en redes sociales**								
0 horas	2.097	47,9	848	25,7	182	62,3	3.127	39,2
1 hora	1.080	24,7	811	24,6	49	16,8	1.940	24,3
2 a 5 horas	1.004	22,9	1.084	32,8	58	19,9	2.146	26,9
6 horas o más	196	4,5	560	17,0	3	1,0	759	9,5
Usa la televisión para acceder a noticias e información sobre COVID-19**								
No	1.002	22,9	624	18,9	160	54,8	1.786	22,4
Sí	3.375	77,1	2.680	81,1	132	45,2	6.187	77,6
**Frecuencia de exposición en la última semana a noticias e información sobre COVID-19 en la televisión**								
Ni una sola vez	601	13,7	394	11,9	41	14,0	1.036	13,0
Pocas veces y algunas veces	2.669	61,0	1.440	43,5	213	72,9	4.322	54,2
A menudo	1.107	25,3	1.473	44,5	38	13,0	2.618	32,8
Horas de exposición a noticias e información sobre COVID-19 en la televisión**								
0 horas	597	13,6	431	13,1	53	18,2	1.081	13,6
1 hora	1.595	36,4	884	26,8	111	38,0	2.590	32,5
2 horas	947	21,6	686	20,8	71	24,3	1.704	21,4
3 horas o más	1.238	28,3	1.301	39,4	57	19,5	2.596	32,5
Usa la radio para acceder a noticias e información sobre COVID-19**								
No	1.866	42,6	2.429	73,5	273	93,5	4.568	57,3
Sí	2.511	57,4	876	26,5	19	6,5	3.406	42,7
**Frecuencia de exposición en la última semana a noticias e información sobre COVID-19 en la radio**								
Ni una sola vez	1.317	30,1	1.956	59,1	211	72,3	3.484	43,7
Pocas veces y algunas veces	2.333	53,3	956	28,9	65	22,3	3.354	42,1
A menudo	727	16,6	395	11,9	16	5,5	1.138	14,3
Horas de exposición a noticias e información sobre COVID-19 en la radio**								
0 horas	1.330	30,4	2.083	63,0	223	76,4	3.636	45,6
1 hora y más	3.047	69,6	1.223	37,0	69	23,6	4.339	54,4

*(n=7.976) **=** Población de estudio; ^†^Muestra total de Perú; ^‡^Muestra total de Brasil; ^§^Muestra total de México; ^||^N = Distribución de los participantes en cada respuesta; ^¶^% = Porcentaje con respecto al total de respuestas en la columna correspondiente a cada variable; **El número total de encuestados es menor que la población total del estudio (7.976)

De las 7.976 personas mayores participantes, 3.395 (42,6%) lograron ser sometidas a pruebas de detección de ansiedad geriátrica, mientras que 4.581 (57,4%) no. En el análisis bivariado de las variables socioeconómicas y demográficas (bloque 2), obtuvieron diferencias significativas, con valor p<0,001, los siguientes ítems: sexo biológico, franja etaria, país de origen, estado civil, raza/color de piel, número de residentes en el hogar, zona de residencia y cambio en los ingresos durante la pandemia de COVID-19. Además, del ítem máximo nivel educativo con valor p=0,032 (Tablas 2 y 3).

**Tabla 2-:** Detección o no de ansiedad y depresión geriátrica y valor p* de las características socioeconómicas y demográficas, y exposición a noticias e información sobre COVID-19 de personas mayores (n^†^ = 7976). Perú, Brasil, México, 2022

**Variables**	**Detección de ansiedad** **geriátrica**				p*	**Detección de depresión** **geriátrica**				p*
	**No**		**Sí**			**No**		**Sí**		
	**N** ^‡^	**%** ^§^	**N** ^‡^	**%** ^§^		**N** ^‡^	**%** ^§^	**N** ^‡^	**%** ^§^	
**Bloque 1 - Variable de origen de los participantes y Bloque 2 - Variables socioeconómicas y demográficas**							
**Sexo biológico**							
Femenino	2.985	65,2	1.956	57,6	<0,001	2.036	62,8	2.905	61,4	0,434
Masculino	1.584	34,6	1.433	42,2		1.199	37,0	1.818	38,4	
Prefiero no declarar	12	0,3	6	0,2		7	0,2	11	0,2	
**País**							
Perú	1.599	34,9	2.778	81,8	<0,001	1.134	35,0	3.243	68,5	<0,001
Brasil	2.712	59,2	595	17,5		1.987	61,3	1.320	27,9	
México	270	5,9	22	0,6		121	3,7	171	3,6	
**Estado civil**							
Soltero(a)	434	9,5	390	11,5	<0,001	352	10,9	472	10,0	<0,001
Casado(a)/En pareja	2.662	58,1	1.980	58,3		1.842	56,8	2.800	59,1	
Divorciado(a)/Separado(a)	552	12,0	302	8,9		432	13,3	422	8,9	
Viudo(a)	933	20,4	723	21,3		616	19,0	1.040	22,0	
**Raza/Color de piel**							
Blanco	2.492	54,4	760	22,4	<0,001	1.674	51,6	1.578	33,3	<0,001
No blanco	2.089	45,6	2.635	77,6		1.568	48,4	3.156	66,7	
**Vivienda propia**							
No	828	18,1	634	18,7	0,501	549	16,9	913	19,3	0,008
Sí	3.753	81,9	2.761	81,3		2.693	83,1	3.821	80,7	
**Zona de residencia**							
Urbana	4.050	88,4	2.780	81,9	<0,001	2.920	90,1	3.910	82,6	<0,001
Rural	531	11,6	615	18,1		322	9,9	824	17,4	
**Bloque 3 - Variables de exposición a noticias e información sobre COVID-19**							
**Usa las redes sociales para acceder a noticias e información sobre COVID-19**							
No	2.300	50,2	1.959	57,7	<0,001	1.613	49,8	2.646	55,9	<0,001
Sí	2.278	49,8	1.436	42,3		1.627	50,2	2.087	44,1	
**Usa la televisión para acceder a noticias e información sobre COVID-19**							
No	1.060	23,2	726	21,4	0,061	690	21,3	1.096	23,2	0,052
Sí	3.518	76,8	2.669	78,6		2.550	78,7	3.637	76,8	
**Usa la radio para acceder a noticias e información sobre COVID-19**							
No	2.877	62,8	1.691	49,8	<0,001	2.111	65,2	2.457	51,9	<0,001
Sí	1.702	37,2	1.704	50,2		1.129	34,8	2.277	48,1	

*p = Chi-cuadrado de Pearson; ^†^Población de estudio; ^‡^N = Distribución de participantes en cada respuesta; ^§^% = Porcentaje respecto al total de respuestas en la columna correspondiente a cada variable

**Tabla 3-:** Detección o no de ansiedad y depresión geriátrica y valor p* de las características socioeconómicas y demográficas, y exposición a noticias e información sobre COVID-19 de personas mayores (n^†^ = 7976). Perú, Brasil, México, 2022

**Variables**	**Detección de ansiedad** **geriátrica**				p*	**Detección de depresión** **geriátrica**				p*
	**No**		**Sí**			**No**		**Sí**		
	**N** ^‡^	**%** ^§^	**N** ^‡^	**%** ^§^		**N** ^‡^	**%** ^§^	**N** ^‡^	**%** ^§^	
**Bloque 1 - Variable de origen de los participantes y Bloque 2 - Variables socioeconómicas y demográficas**										
**Franja etaria (años)**										
60 – 64	1.570	34,3	1.017	30,0	<0,001	1.191	36,7	1.396	29,5	<0,001
65 – 69	1.303	28,4	988	29,1		889	27,4	1.402	29,6	
70 – 74	730	15,9	538	15,8	<0,001	514	15,9	754	15,9	<0,001
75 – 79	507	11,1	417	12,3		358	11,0	566	12,0	
80 o más	471	10,3	435	12,8		290	8,9	616	13,0	
**Número de residentes en el hogar**										
Vive solo	569	12,4	240	7,1	<0,001	427	13,2	382	8,1	<0,001
Una o dos personas	2.155	47,0	1.268	37,3		1.520	46,9	1.903	40,2	
Tres o más personas	1.857	40,5	1.887	55,6		1.295	39,9	2.449	51,7	
**Máximo nivel educativo**										
No estudió o no completó la educación básica	403	8,8	272	8,0	0,032	234	7,2	441	9,3	<0,001
Educación básica o primaria	1.163	25,4	799	23,5		751	23,2	1.211	25,6	
Educación secundaria	1.135	24,8	944	27,8		807	24,9	1.272	26,9	
Educación superior completa	1.029	22,5	1.091	32,1		784	24,2	1.336	28,2	
Otros	851	18,6	289	8,5		666	20,5	474	10,0	
**Cambios en los ingresos durante la pandemia de COVID-19**										
No	2.564	56,0	1.548	45,6	<0,001	1.977	61,0	2.135	45,1	<0,001
Sí, mis ingresos aumentaron	149	3,3	118	3,5		105	3,2	162	3,4	
Sí, mis ingresos disminuyeron	1.866	40,8	1.728	50,9		1.158	35,7	2.436	51,5	
**Bloque 3 - Variables de exposición a noticias e información sobre COVID-19**										
**Frecuencia de exposición en la última semana a noticias e información sobre COVID-19 en redes sociales**										
Ni una sola vez	1.686	36,8	1.575	46,4	<0,001	1.146	35,3	2.115	44,7	<0,001
Pocas veces y algunas veces	1.913	41,8	1.224	36,1		1.325	40,9	1.812	38,3	
A menudo	982	21,4	596	17,6		771	23,8	807	17,0	
**Horas de exposición a noticias e información sobre COVID-19 en redes sociales**										
0 horas	1.635	35,7	1.492	43,9	<0,001	1.118	34,5	2.009	42,4	<0,001
1 hora	1.059	23,1	881	25,9		836	25,8	1.104	23,3	
2 a 5 horas	1.368	29,9	778	22,9		930	28,7	1.216	25,7	
6 horas o más	515	11,3	244	7,2		355	11,0	404	8,	
**Frecuencia de exposición en la última semana a noticias e información sobre COVID-19 en la televisión**										
Ni una sola vez	572	12,5	464	13,7	<0,001	421	13,0	615	13,0	<0,001
Pocas veces y algunas veces	2.414	52,7	1.908	56,2		1.662	51,3	2.660	56,2	
A menudo	1.595	34,8	1023	30,1		1.159	35,7	1.459	30,8	
**Horas de exposición a noticias e información sobre COVID-19 en la televisión**										
0 horas	590	12,9	491	14,5	0,012	479	14,8	602	12,7	0.075
1 hora	1.501	32,8	1.089	32,1		1.028	31,7	1.562	33,0	
2 horas	924	20,2	780	23,0		708	21,9	996	21,0	
3 horas o más	1.561	34,1	1.035	30,5		1.024	31,6	1.572	33,2	
**Frecuencia de exposición en la última semana a noticias e información sobre COVID-19 en la radio**										
Ni una sola vez	2.180	47,6	1.304	38,4	<0,001	1.656	51,1	1.828	38,6	<0,001
Pocas veces y algunas veces	1.838	40,1	1.516	44,7		1.190	36,7	2.164	45,7	
A menudo	563	12,3	575	16,9		396	12,2	742	15,7	
**Horas de exposición a noticias e información sobre COVID-19 en la radio**										
0 horas	2.295	50,1	1.341	39,5	<0,001	1.779	54,9	1.857	39,2	<0,001
1 hora y más	2.285	49,9	2.054	60,5		1.462	45,1	2.877	60,8	

*p = Prueba U de Mann-Whitney; ^†^Población de estudio; ^‡^N = Distribución de participantes en cada respuesta; ^§^% = Porcentaje respecto al total de respuestas en la columna correspondiente a cada variable

En la asociación de la variable detección/no detección de ansiedad con las variables de exposición a noticias e información (bloque 3), se observaron diferencias significativas en los ítems: uso, frecuencia y horas de exposición a través de redes sociales para acceder a noticias e información sobre COVID-19 con valor p<0,001. Para uso, frecuencia y horas de exposición a la radio para acceder a noticias e información sobre COVID-19 con valor p<0,001. Con respecto a la televisión, sólo las variables frecuencia y horas de exposición resultaron significativas, con valor p<0,001 y valor p=0,012, respectivamente.

En cuanto a la detección de depresión geriátrica, 4.734 (59,4%) indicaron detección, mientras que 3.242 (40,6%) no. Las siguientes variables socioeconómicas y demográficas (bloques 1 y 3) fueron significativas en el análisis bivariado: franja etaria, país de origen, estado civil, raza/color de piel, número de residentes en el hogar, zona de residencia, máximo nivel educativo y cambio en los ingresos durante la pandemia de COVID-19, con valor p<0,001 para cada ítem. Sólo el ítem vivienda propia, entre las diferencias significativas, alcanzó valor p=0,008.

Del análisis de asociación bivariada entre detección/no detección de depresión geriátrica y las variables de exposición a noticias e información (bloque 3), resultaron estadísticamente significativos los ítems: uso, frecuencia y horas de exposición a través de redes sociales para acceder a noticias e información sobre COVID-19 con valor p<0,001 para cada ítem. Para uso, frecuencia y horas de exposición a la radio para acceder a noticias e información sobre COVID-19 con valor p<0,001, respectivamente. Respecto a la TV, sólo el ítem frecuencia fue significativo, con valor p<0,001.

En el análisis multivariado, las siguientes variables presentaron valor p<0,05 en la regresión independiente de los bloques, para la detección de ansiedad: bloque 1, país de origen; bloque 2, sexo, franja etaria, estado civil, raza/color de piel (no blanco), número de residentes en el hogar, zona de residencia, educación (secundaria o superior) y cambio en los ingresos durante la pandemia de COVID-19 (disminución de los ingresos); bloque 3, frecuencia (a menudo) y horas (dos horas o más) de exposición a noticias e información sobre COVID-19 en redes sociales, uso y horas (una hora) de exposición a noticias e información sobre COVID-19 en televisión, y uso, frecuencia (a menudo) y ni una hora de exposición a noticias e información sobre COVID-19 en la radio.

Sin embargo, en el modelo final (Tabla 4), con R² de Nagelkerke=0,312 y valor p<0,001 de la prueba de Hosmer y Lemeshow, las siguientes variables siguieron siendo significativas: país de origen, sexo (masculino), estado civil, raza (no blanca), cambio en los ingresos durante la pandemia de COVID-19, frecuencia (a menudo) y horas (dos horas o más) de exposición a noticias e información sobre COVID-19 en redes sociales, uso de la televisión y horas (una hora) para acceder a noticias e información sobre COVID-19, y frecuencia (algunas veces) de exposición a noticias e información sobre COVID-19 en la radio.

Para la detección de depresión, en la regresión independiente de los bloques, las variables que presentaron diferencias estadísticamente significativas fueron: bloque 1, país de origen; bloque 2, sexo (masculino), franja etaria, estado civil (divorciado/separado), raza/color de piel (no blanco), número de residentes en el hogar, zona de residencia, máximo nivel educativo (otros) y cambio en los ingresos durante la pandemia de COVID-19 (disminución de los ingresos); bloque 3, uso, frecuencia (algunas veces y a menudo) y horas (una hora o más) de exposición a noticias e información sobre COVID-19 en redes sociales, uso y horas (una hora o más) de exposición a noticias e información sobre COVID-19 en televisión, y uso y horas (una hora o más) de exposición a noticias e información sobre COVID-19 en la radio.

**Tabla 4-:** Modelo final de detección o no de ansiedad geriátrica y valor p* de las características socioeconómicas, demográficas y exposición a noticias e información sobre COVID-19 de personas mayores (n^†^ = 7.976). Perú, Brasil, México, 2022

**Variables**	**Exposición (B) ajustado en el** **bloque (IC** **95%)** ^‡^		p*		**Exposición (B) ajustado en el modelo final (IC 95%)** ^‡^			p*
**Bloque 1 - Variable de origen de los participantes**								
**País**								
Perú	Ref ^§^							
Brasil	0,13 0,11-0,14		<0,001		0,10 0,09-0,12			<0,001
México	0,05 0,03-0,07		<0,001		0,05 0,03-0,08			<0,001
**Bloque 2 - Variables socioeconómicas y demográficas**								
**Sexo biológico**								
Femenino		Ref ^§^						
Masculino		1,13 1,02-1,25		0,018		1,13 1,01-1,26	0,031	
Prefiero no declarar		0,85 0,31-2,38		0,761		2,46 0,90-6,72	0,079	
**Franja etaria (años)**								
60-64		Ref ^§^						
65-69		1,14 1,00-1,29		0,043		1,07 0,94-1,23	0,300	
70-74		1,18 1,02-1,37		0,031		0,99 0,84-1,16	0,900	
75-79		1,26 1,07-1,49		0,006		1,05 0,88-1,27	0,577	
80 o más		1,45 1,22-1,73		<0,001		1,04 0,86-1,26	0,690	
**Estado civil**								
Soltero(a)		Ref ^§^						
Casado(a)/en pareja		0,61 0,52-0,73		<0,001		0,68 0,57-0,82	<0,001	
Divorciado(a)/separado(a)		0,63 0,51-0,78		<0,001		0,74 0,59-0,93	0,009	
Viudo(a)		0,70 0,58-0,84		<0,001		0,72 0,59-0,89	0,002	
**Raza/Color de piel**								
Blanco		Ref ^§^						
No blanco		3,63 3,26-4,05		<0,001		1,39 1,21-1,59	<0,001	
**Número de residentes en el hogar**								
Vive solo		Ref ^§^						
Uno o dos		1,26 1,04-1,51		0,017		1,06 0,87-1,30	0,563	
Tres o más		1,76 1,45-2,12		<0,001		1,02 0,82-1,25	0,886	
**Vivienda propia**								
No		Ref ^§^						
Sí		1,10 0,97-1,24		0,146		1,13 0,99-1,30	0,073	
**Zona de residencia**								
Urbana		Ref ^§^						
Rural		1,34 1,17-1,54		<0,001		0,92 2,79-1,06	0,257	
**Máximo nivel educativo**								
No estudió o no completó la educación básica		Ref ^§^						
Educación básica o primaria		1,15 0,95-1,39		0,139		0,92 0,75-1,13	0,420	
Educación secundaria		1,60 1,32-1,94		<0,001		1,04 0,83-1,28	0,754	
Educación superior completa		2,21 1,82-2,68		<0,001		1,22 0,97-1,53	0,082	
Otros		1,28 1,01-1,61		0,039		1,21 0,94-1,58	0,144	
**Cambios en los ingresos durante la pandemia de COVID-19**								
No		Ref ^§^						
Sí, mis ingresos aumentaron		1,06 0,81-1,39		0,671		0,71 0,53-0,95	0,021	
Sí, mis ingresos disminuyeron		1,12 1,01-1,24		0,029		0,72 0,64-0,81	<0,001	
**Bloque 3 - Variables de exposición a noticias e información sobre COVID-19**								
**Usa las redes sociales para acceder a noticias e información sobre COVID-19**								
No		Ref ^§^						
Sí		1,05 0,92-1,20		0,448		0,98 0,85-1,14	0,819	
**Frecuencia de exposición en la última semana a noticias e información sobre COVID-19 en redes sociales**								
Ni una sola vez		Ref ^§^						
Pocas veces y algunas veces		0,77 0,65-0,91		0,002		1,13 0,93-1,36	0,217	
A menudo		0,94 0,76-1,15		0,528		1,59 1,25-2,03	<0,001	
**Horas de exposición a noticias e información sobre COVID-19 en redes sociales**								
0 horas		Ref ^§^						
1 hora		1,14 0,96-1,35		0,137		1,04 0,85-1,26	0,710	
2 a 5 horas		0,77 0,64-0,92		0,004		0,73 0,59-0,90	0,003	
6 horas o más		0,62 0,49-0,78		<0,001		0,87 0,67-1,14	0,324	
**Usa la televisión para acceder a noticias e información sobre COVID-19**								
No		Ref ^§^						
Sí		1,20 1,04-1,39		0,010		1,20 1,02-1,41	0,031	
**Frecuencia de exposición en la última semana a noticias e información sobre COVID-19 en la televisión**								
Ni una sola vez		Ref ^§^						
Pocas veces y algunas veces		1,07 0,88-1,30		0,492		0,97 0,78-1,21	0,810	
A menudo		0,84 0,67-1,04		0,112		0,92 0,71-1,18	0,502	
**Horas de exposición a noticias e información sobre COVID-19 en la televisión**								
0 horas		Ref ^§^						
1 hora		0,78 0,64-0,95		0,013		0,70 0,56-0,88	0,002	
2 horas		0,98 0,79-1,21		0,818		0,97 0,76-1,23	0,782	
3 horas o más		0,83 0,67-1,03		0,094		0,84 0,66-1,07	0,162	
**Usa la radio para acceder a noticias e información sobre COVID-19**								
No		Ref ^§^						
Sí		1,30 1,14-1,48		<0,001		1,03 0,88-1,20	0,724	
**Frecuencia de exposición en la última semana a noticias e información sobre COVID-19 en la radio**								
Ni una sola vez		Ref ^§^						
Pocas veces y algunas veces		1,01 0,87-1,18		0,852		0,77 0,65-0,91	0,003	
A menudo		1,26 1,04-1,53		0,018		1,06 0,85-1,32	0,615	
**Horas de exposición a noticias e información sobre COVID-19 en la radio**								
0 horas		Ref ^§^						
1 hora y más		1,22 1,05-1,42		0,010		0,85 0,71-1,01	0,067	

*p = Valor p; ^†^Población de estudio; ^‡^(IC 95%) = Intervalo de confianza del 95%; ^§^Ref = Categoría de referencia

En el modelo final (Tabla 5), con R² de Nagelkerke=0,169 y valor p<0,001 de la prueba de Hosmer y Lemeshow, las siguientes variables presentaron valor p<0,05: país de origen, sexo (masculino), franja etaria (80 años o más), raza (no blanca), educación (de educación básica o primaria en adelante), cambio en los ingresos durante la pandemia de COVID-19 (disminución de los ingresos), uso y horas (una hora o más) de exposición a noticias e información sobre COVID-19 en la televisión, y horas (una hora o más) de exposición a noticias e información sobre COVID-19 en la radio.

**Tabla 5-:** Modelo final de detección o no de depresión geriátrica y valor p* de las características socioeconómicas, demográficas y exposición a noticias e información sobre COVID-19 de las personas mayores (n^†^ = 7.976). Perú, Brasil, México, 2022

**Variables**	**Exposición (B) ajustado en el bloque (IC** **95%)** ^‡^	p*	**Exposición (B) ajustado en el modelo final (IC 95%)** ^‡^			p*
**Bloque 1 - Variable de origen de los participantes**						
**País**						
Perú	Ref ^§^					
Brasil	0,23 0,21-0,26	<0,001	0,23 0,19-0,26			<0,001
México	0,49 0,39-0,63	<0,001	0,45 0,34-0,60			<0,001
**Bloque 2 - Variables socioeconómicas y demográficas**						
**Sexo biológico**						
Femenino	Ref ^§^					
Masculino	0,88 0,80-0,98	0,014		0,83 0,75-0,92	0,001	
Prefiero no declarar	1,29 0,49-3,41	0,610		2,07 0,79-5,44	0,138	
**Franja etaria (años)**						
60-64	Ref ^§^					
65-69	1,26 1,12-1,43	<0,001		1,17 1,04-1,33	0,012	
70-74	1,26 1,09-1,46	0,002		1,09 0,94-1,27	0,241	
75-79	1,29 1,09-1,51	0,003		1,15 0,97-1,37	0,110	
80 o más	1,70 1,43-2,03	<0,001		1,43 1,18-1,72	<0,001	
**Estado civil**						
Soltero(a)	Ref ^§^					
Casado(a)/en pareja	0,98 0,83-1,15	0,775		1,08 0,91-1,28	0,405	
Divorciado(a)/separado(a)	0,77 0,63-0,94	0,010		0,87 0,71-1,07	0,200	
Viudo(a)	1,02 0,85-1,22	0,864		1,05 0,86-1,26	0,647	
**Raza/Color de piel**						
Blanco	Ref ^§^					
No blanco	1,62 1,47-1,80	<0,001		0,86 0,76-0,98	0,022	
**Número de residentes en el hogar**						
Vive solo	Ref ^§^					
Uno o dos	1,19 1,01-1,41	0,043		1,05 0,88-1,25	0,583	
Tres o más	1,46 1,22-1,74	<0,001		0,96 0,80-1,15	0,655	
**Vivienda propia**						
No	Ref ^§^					
Sí	0,93 0,82-1,05	0,259		0,93 0,81-1,05	0,246	
**Zona de residencia**						
Urbana	Ref ^§^					
Rural	1,46 1,26-1,69	<0,001		1,09 0,93-1,27	0,283	
**Máximo nivel educativo**						
No estudió o no completó la educación básica	Ref ^§^					
Educación básica o primaria	0,91 0,75-1,10	0,337		0,76 0,62-0,93	0,007	
Educación secundaria	1,00 0,82-1,21	0,990		0,71 0,58-0,88	0,001	
Educación superior completa	1,12 0,92-1,36	0,246		0,71 0,57-0,88	0,002	
Otros	0,70 0,56-0,87	0,001		0,66 0,52-0,83	<0,001	
**Cambios en los ingresos durante la pandemia de COVID-19**						
No	Ref ^§^					
Sí, mis ingresos aumentaron.	1,26 0,97-1,63	0,084		0,97 0,74-1,27	0,815	
Sí, mis ingresos disminuyeron.	1,66 150-1,83	<0,001		1,19 1,07-1,33	0,001	
**Bloque 3 - Variables de exposición a noticias e información sobre COVID-19**						
**Usa las redes sociales para acceder a noticias e información sobre COVID-19**						
No	Ref ^§^					
Sí	1,15 1,01-1,31	0,037		1,13 0,99-1,30	0,078	
**Frecuencia de exposición en la última semana a noticias e información sobre COVID-19 en redes sociales**						
Ni una sola vez	Ref ^§^					
Pocas veces y algunas veces	0,81 0,69-0,96	0,015		1,12 0,94-1,34	0,216	
A menudo	0,68 0,55-0,84	<0,001		1,00 0,80-1,26	0,968	
**Horas de exposición a noticias e información sobre COVID-19 en redes sociales**						
0 horas	Ref ^§^					
1 hora	0,82 0,69-0,98	0,026		0,86 0,71-1,04	0,114	
2 a 5 horas	0,82 0,68-0,98	0,031		0,92 0,76-1,12	0,398	
6 horas o más	0,76 0,60-0,95	0,017		1,08 0,85-1,38	0,529	
**Usa la televisión para acceder a noticias e información sobre COVID-19**						
No	Ref ^§^					
Sí	0,79 0,68-0,91	0,001		0,86 0,73-1,00	0,047	
**Frecuencia de exposición en la última semana a noticias e información sobre COVID-19 en la televisión**						
Ni una sola vez	Ref ^§^					
Pocas veces y algunas veces	1,01 0,83-1,23	0,940		0,91 0,74-1,12	0,362	
A menudo	0,88 0,70-1,09	0,244		0,94 0,74-1,19	0,590	
**Horas de exposición a noticias e información sobre COVID-19 en la televisión**						
0 horas	Ref ^§^					
1 hora	1,44 1,18-1,76	<0,001		1,36 1,10-1,68	0,005	
2 horas	1,41 1,13-1,75	0,002		1,36 1,09-1,72	0,008	
3 horas o más	1,59 1,28-1,97	<0,001		1,66 1,32-2,09	<0,001	
**Usa la radio para acceder a noticias e información sobre COVID-19**						
No	Ref ^§^					
Sí	1,23 1,08-1,41	0,002		1,08 0,93-1,25	0,305	
**Frecuencia de exposición en la última semana a noticias e información sobre COVID-19 en la radio**						
Ni una sola vez	Ref ^§^					
Pocas veces y algunas veces	1,05 0,90-1,22	0,541		0,90 0,77-1,06	0,217	
A menudo	1,06 0,87-1,29	0,539		0,98 0,80-1,21	0,868	
**Horas de exposición a noticias e información sobre COVID-19 en la radio**						
0 horas	Ref ^§^					
1 hora y más	1,49 1,28-1,74	<0,001		1,21 1,03-1,43	0,020	

*p = Valor p; ^†^Población de estudio; ^‡^(IC 95%) = Intervalo de confianza del 95%; ^§^Ref = Categoría de referencia

## Discusión

Este estudio analizó la asociación entre la exposición a noticias e información sobre COVID-19 y el impacto en la salud mental de los adultos mayores en Perú, Brasil y México. La prevalencia de detección de ansiedad geriátrica entre los participantes fue del 42,6%. Cabe destacar que, sólo en la muestra de adultos mayores de Perú, la prevalencia fue mayoritaria para el resultado, con 63,5%. Se detectaron síntomas de depresión en el 59,4% de la muestra y la frecuencia fue de 74,1% en las muestras de participantes peruanos y 58,6% en la muestra mexicana.

Los hallazgos están por encima de la frecuencia observada en una revisión sistemática^(13)^ centrada en América Latina, que estima una prevalencia del 35% (IC 95%: 31%-38%) para ansiedad y 35% (IC 95%: 31%-39%) para síntomas de depresión. En otras regiones del mundo^(11)^, la prevalencia de los síntomas de ansiedad varía de 7,4% a 47,82% y los de depresión de 14,14% a 48,3%. Sin embargo, cabe aclarar que las revisiones no cuentan con una muestra compuesta exclusivamente por personas mayores y los instrumentos utilizados para la detección de síntomas difieren de los aplicados en el estudio. Además, el inicio del período de recolección de datos coincidió con el primer pico más alto de muertes por COVID-19 registrado en las Américas, principalmente el 18 de enero de 2021, lo que también puede haber contribuido a los distintos hallazgos presentes en la literatura^(1)^.

Con respecto al uso de medios de comunicación para acceder a noticias e información sobre COVID-19, el 77,6% de los participantes indicó que lo hizo mediante la televisión, el 46,6% que usó redes sociales y el 42,7% recurrió a la radio. Sólo en la muestra de adultos mayores brasileños, se observó que prevalencia el uso de redes sociales, con 58,8%. Sólo en la muestra de participantes peruanos predominó el uso de la radio, con 57,4%. Los participantes mexicanos se destacan por no utilizar la televisión, con una frecuencia de 54,8%, además de no presentar una frecuencia predominantemente positiva en ninguno de los tres medios.

Se puede observar que no hubo concordancia entre los tres países sobre ninguno de los ítems. Un análisis del periodismo mundial a inicios de 2023, indica que el acceso a internet en Perú, Brasil y México es del 87%, 83% y 67%, respectivamente, y que el acceso *online* (incluyendo redes sociales) es la principal fuente de acceso a noticias, aunque se percibe una disminución generalizada de dicho consumo. Esa reducción también se identifica en los medios tradicionales, como la televisión y los periódicos impresos^(23)^.

En Brasil, el consumo de noticias *online* (incluyendo redes sociales) disminuyó del 90% al 79% en los últimos 10 años, especialmente el uso de *WhatsApp*. El 51% de los participantes mencionó la televisión. De forma similar, el consumo en Perú y México de noticias *online* (incluyendo redes sociales) es del 80%, principalmente a través de *Facebook*. La diferencia radica en el consumo de la televisión, que es del 51% en Perú y del 42% en México^(23)^.

Distinguir los hallazgos entre países nos inspira a enfatizar el contexto de esos territorios y a comprender el comportamiento informacional de la muestra poblacional compuesta exclusivamente por personas mayores. Se observa que los participantes buscaron noticias e información sobre COVID-19, incluso a través de medios digitales (redes sociales). Sin embargo, no se midió la calidad de la información, el nivel de confianza ni el nivel de alfabetización de las personas mayores.

En un estudio prepandemia sobre el acceso a los medios digitales de las personas mayores en algunos países de América Latina, incluidos Perú y México, se observó que las personas mayores representaban menos del 10% de la población de usuarios, y que predomina el uso para obtener información, seguido de la comunicación^(24)^. Durante la pandemia^(8)^, indican que en Perú la incapacidad para reconocer noticias falsas alcanzó al 79% de la población. Por encima de México, con 66%, y Brasil, con 62%.

Esto se combina con baja alfabetización digital de las personas mayores^(25)^ y niveles decrecientes de confianza pública en las noticias, principalmente debido a la situación política y el comportamiento de sus presidentes, a saber, Brasil con un 43% de confianza, seguido de México con un 36% y Perú con un 33%^(23)^.

En nuestra muestra, la asociación bivariada del uso de medios para el resultado detección de ansiedad presentó diferencias estadísticamente significativas en el uso de redes sociales (X²=43,6 y valor p<0,001) y radio (X²=135,0 y valor p<0,001). Sin embargo, sólo en el caso de la radio había una frecuencia del 50% de que el resultado se produjera o no. Con respecto a la detección de depresión, para las redes sociales (X²=29,0 y valor p<0,001), la televisión (X²=3,83 y valor p<0,050) y la radio (X²=138,0 y valor p< 0,001) la frecuencia del resultado fue mayor independientemente del uso.

Una revisión integradora analizó el impacto de las redes sociales digitales en la percepción de soledad y aislamiento social en las personas mayores e indicó que el uso de estos medios tiene el potencial de reducir los sentimientos de soledad y aislamiento social y de promover una mayor interacción y un sentido de pertenencia en las personas mayores^(26)^.

Esos hallazgos coinciden parcialmente con los resultados del presente estudio, ya que también identificamos una asociación significativa entre la exposición a las redes sociales y los cambios en la salud mental de los participantes durante la pandemia. Sin embargo, a diferencia de los resultados encontrados en la revisión integradora, nuestro estudio indicó que la exposición frecuente a las redes sociales se asoció con una mayor prevalencia de síntomas de ansiedad y depresión, lo que sugiere que el contexto de la pandemia puede haber influido negativamente en esa relación^(26)^.

Por lo tanto, los hallazgos señalan a las personas mayores que consumen información sobre COVID-19 y en las que se pueden detectar trastornos mentales, especialmente depresión, pero que, dentro de los límites del método y los análisis, no es posible inferir la dirección de las reacciones considerando el contexto conocido de los participantes.

En el modelo final de detección de ansiedad geriátrica, con una variación de 31,18% explicada por el R² de Nagelkerke y el valor p<0,001 de Hosmer y Lemeshow, controlado por factores socioeconómicos, se estima que los adultos mayores participantes en Brasil tuvieron 0,10 (IC 95%: 0,09-0,12 y valor p<0,001) veces la probabilidad de detección de trastornos de ansiedad y los adultos mayores en México, 0,05 (IC 95%: 0,03-0,08 y valor p<0,001) veces la probabilidad, en función de la categoría de referencia, adultos mayores de Perú.

Con respecto a la exposición a noticias e información sobre COVID-19, los tres medios presentaron diferente significación. El uso de redes sociales con este fin indicó 1,59 (IC 95%: 1,25-2,03 y valor p<0,001) veces la probabilidad de detección de ansiedad geriátrica en relación a quienes no lo utilizaron de esta manera. De forma similar, pasar de 2 a 5 horas en redes sociales presentó 0,73 (IC 95%: 0,59-0,90 y valor p=0,003) veces las posibilidades de detección en comparación con quienes no estuvieron expuestos en ningún momento.

En cuanto a la televisión, el uso para informarse sobre COVID-19 supuso 1,20 (IC 95%: 1,02-1,41 y valor p=0,031) veces la probabilidad de que se produzca el resultado en comparación con quienes no la utilizan. Los expuestos a una hora tienen 0,70 (IC 95%: 0,56-0,88 y valor p=0,002) veces la probabilidad de detección en comparación con quienes no tienen ninguna hora de exposición con esta intención. La radio, solo la frecuencia, una o dos veces por semana, indica 0,77 (IC 95%: 0,65-0,91 y valor p=0,003) veces más probabilidad del resultado que aquellos que no estuvieron expuestos a información sobre COVID-19.

En el segundo modelo, con resultado depresión y una variación explicada del 16,89% según R² de Nagelkerke y valor p<0,001 de Hosmer y Lemeshow, controlado por factores socioeconómicos, los brasileños presentaron 0,23 (IC 95%: 0,19-0,26 y valor p<0,001) veces la probabilidad de detección y los mexicanos 0,45 (IC 95%: 0,34-0,60 y valor p<0,001) veces la probabilidad de detección, con respecto a los adultos mayores peruanos.

Sólo en la televisión y la radio se observó significación en el resultado. Tres horas o más de exposición a noticias e información sobre COVID-19 en la televisión arrojó 1,66 (IC 95%: 1,32-2,09 y valor p<0,001) veces más que quienes no se expusieron ninguna hora para ello. El tiempo de exposición también fue relevante en la radio, una hora o más indicó 1,21 (IC 95%: 1,03-1,43 y valor p=0,020) veces más en comparación con quienes no consumían este medio.

Estos resultados reafirman la asociación entre consumo de medios (digitales o no) de información sobre COVID-19 y la posibilidad de detectar los trastornos mentales analizados en participantes de edad avanzada. La ausencia o el bajo consumo de alguno de los medios no indicó mayor probabilidad de obtener resultados en ninguna de las variables asociadas.

En una revisión sistemática^(27)^, el uso de las redes sociales es una de las causas de la infodemia y entre los impactos se destacan los problemas psicológicos. El ambiente propicio para la circulación de desinformación (de diversas maneras) fueron las redes sociales y, inevitablemente, estuvieron desbordadas. En un “círculo vicioso de infodemia”^(27)^ el impacto en la salud mental amplifica la maquinaria infodémica que se manifiesta nuevamente en forma de trastornos que empeoran.

En el movimiento de gestión de la infodemia se discute la desigualdad en el acceso a información de calidad, actualizada y acorde a las necesidades de las personas y las comunidades. En términos de daño potencial a la salud, los estados emocionales, que derivan de una sobrecarga de información, pueden no desencadenarse debido a la información, sino derivar de estados previamente existentes, que pueden influir en el comportamiento de los individuos^(6)^.

Debido a los límites que impone el objetivo del artículo, no es posible abarcar todos los matices de la complejidad de la información para la salud, pero se suma la discusión^(28)^ de que la sobrecarga de información no es un proceso agudo, sino estructural, que forma parte de la determinación social de la salud. La desigualdad informativa, la infodemia, el edadismo, los monopolios mediáticos y el modelo manicomial de salud operan en el mismo universo de comercialización de la vida. La ruptura de esas estructuras prevé no sólo el antagonismo, sino la construcción de un proyecto de sociedad que incluya también la garantía del derecho a una información de calidad, gratuita, segura y accesible.

Una limitación del estudio radica en el esfuerzo por utilizar los mismos instrumentos de detección de ansiedad y depresión geriátrica en los tres países, lo que puede haber influido en la prevalencia. Además, como es típico de los estudios observacionales transversales, no se puede determinar si la relación cronológica entre la recolección de datos y la pandemia de COVID-19 modificaría los hallazgos. También somos conscientes de la posibilidad de que existan sesgos de selección e información en la recolección de datos mediante *web-based survey*, en una muestra no probabilística, lo que restringe la generalización de los hallazgos.

Este estudio contribuye significativamente al avance científico al demostrar que hay diferencias transnacionales en la prevalencia de ansiedad y depresión geriátricas asociadas al consumo de medios durante la pandemia en adultos mayores en Perú, Brasil y México. Los hallazgos son relevantes para la práctica de la enfermería gerontológica en esos países, ya que orientan estrategias específicas para promover la salud mental y la alfabetización en salud adaptadas a las realidades locales.

## Conclusión

Se encontró una asociación entre la exposición frecuente a los medios de comunicación y una mayor prevalencia de ansiedad y depresión geriátrica en los adultos mayores estudiados. La diferencia significativa en la prevalencia del resultado, al comparar los tres países, fue pequeña. Estos resultados enfatizan que es necesario crear estrategias específicas para el manejo de la infodemia a fin de proteger la salud mental de los adultos mayores en América Latina.

Dadas las limitaciones del estudio, las personas mayores en los países latinoamericanos analizados coinciden con lo descrito en la literatura, que indica que dicha población está presente y adopta medios digitales (en este estudio, enfocado en redes sociales). Además, la salud mental de los ancianos, que se extiende a las relaciones con la información, confirma los hallazgos que indican que demandan y necesitan cuidados y políticas públicas focalizadas. Por lo tanto, se destaca que no se pretende delimitar y reforzar estereotipos biomédicos sobre la experiencia y relación de los individuos con los trastornos mentales, ya que una posible sintomatología de manera aislada no determina el comportamiento y las elecciones de los individuos.

La investigación nos permite contribuir al conocimiento producido y centrado en América Latina, con articulación actual, vinculado a la pandemia de COVID-19 y a las implicaciones futuras, articulándolo al envejecimiento poblacional y a la gestión de la infodemia, así como a las políticas de salud pública. Se espera contribuir a investigaciones que incluyan el derecho a la información y el envejecimiento saludable e invertir en prevención y promoción de la salud a través de la educación –alfabetización en salud– y de la participación –escuchar las demandas y necesidades de salud– para crear y/o fortalecer políticas públicas intersectoriales conscientes del poder de los pueblos latinoamericanos.
